# Genetic heterogeneity on sleep disorders in Parkinson’s disease: a systematic review and meta-analysis

**DOI:** 10.1186/s40035-022-00294-1

**Published:** 2022-04-08

**Authors:** Jingxuan Huang, Yangfan Cheng, Chunyu Li, Huifang Shang

**Affiliations:** Laboratory of Neurodegenerative Disorders, Department of Neurology, Rare Diseases Center, National Clinical Research Center for Geriatrics, West China Hospital, Sichuan University, Chengdu, 610041 China

**Keywords:** Genetic variants, Sleep disorder, Rapid-eye-movement behavior disorders, *GBA*, *LRRK2*

## Abstract

A growing amount of evidence has indicated contributions of variants in causative genes of Parkinson’s disease (PD) to the development of sleep disturbance in PD and prodromal PD stages. In this article, we aimed to investigate the role of genetics in sleep disorders in PD patients and asymptomatic carriers at prodromal stage of PD. A systematic review and meta-analysis of observational studies was conducted based on the MEDLINE, EMBASE and PsychINFO databases. A pooled effect size was calculated by odds ratio (OR) and standard mean difference (SMD). Forty studies were selected for quantitative analysis, including 17 studies on glucocerebrosidase (*GBA*), 25 studies on Leucine-rich repeat kinase 2 (*LRRK2*) and 7 on parkin (*PRKN*) genes, and 3 studies on alpha-synuclein gene (*SNCA*) were used for qualitative analysis. Patients with PD carrying *GBA* variants had a significantly higher risk for rapid-eye-movement behavior disorders (RBD) (OR, 1.82) and higher RBD Screening Questionnaire scores (SMD, 0.33). Asymptomatic carriers of *GBA* variants had higher severity of RBD during follow-up. Patients with PD carrying the *LRRK2* G2019S variant had lower risk and severity of RBD compared with those without *LRRK2* G2019S. Variants of *GBA*, *LRRK2* and *PRKN* did not increase or decrease the risk and severity of excessive daytime sleepiness and restless legs syndrome in PD. Our findings suggest that the genetic heterogeneity plays a role in the development of sleep disorders, mainly RBD, in PD and the prodromal stage of PD.

**Supplementary Information:**

The online version contains supplementary material available at 10.1186/s40035-022-00294-1.

## Background

Parkinson’s disease (PD) is one of the most common neurodegenerative disorders, manifesting mainly as bradykinesia, resting tremor and various non-motor symptoms. Increasing evidence has witnessed that the non-motor symptoms have a significant impact on the quality of life of patients with PD. The non-motor symptoms can occur either after motor onset or before motor symptoms at a prodromal stage [1]. Sleep disorders are common non-motor symptoms, including excessive daytime sleepiness (EDS), insomnia, restless legs syndrome (RLS), circadian rhythm disorders, sleep attacks, obstructive sleep apnea and rapid-eye-movement behavior disorders (RBD). In addition, some sleep disorders such as RBD are considered as a prodrome of α-synucleinopathies [2], suggesting that sleep disorders are closely related to the pathophysiology of PD.

Currently, multiple variants of genes, such as alpha-synuclein gene (*SNCA*), leucine-rich repeat kinase 2 gene (*LRRK2*), glucocerebrosidase gene (*GBA*) and parkin gene (*PRKN*), have been reported to be causes of PD. Different variants in such causative genes also play a role in the discrepancies in clinical manifestations and prognosis of PD. As one of the most common non-motor symptoms of PD, sleep disorders are strongly associated with some genes, since a growing number of genome-wide association studies have identified genetic risks for sleep disorders [3]. Therefore, sleep disorders in patients carrying PD-related gene mutations have received much attention for research. However, studies have not reached a consensus regarding the genetic risk factors for sleep disorders in PD or prodromal PD. Some studies have found that PD patients carrying *GBA* variants seem to develop RBD more frequently than patients without *GBA* variants [4]. However, another cohort study did not find any difference in the risk of RBD between patients with and without *GBA* variants [5]. Inconsistent results have been found on the prevalence and the severity of other sleep disorders, such as EDS and RLS, from studies on *LRRK2* [6] or *PRKN* variants [7, 8]. Moreover, controversial conclusions regarding the role of genetic variants on sleep disorders exist when comparing asymptomatic carriers of causative gene variants with non-carrier healthy controls (HCs) [9, 10]. Differences in the sites of variants of causative genes, disease durations of participants in cohorts, and the study design could contribute to such discrepancies. Therefore, the association of genetic heterogeneity with the risk of sleep disorders may differ, especially at different stages of PD, such as prodromal and clinical stages.

In this context, we systematically reviewed the genetic variants associated with the risk of sleep disorders in PD patients and asymptomatic PD genetic carriers to elucidate this inconsistency.

## Methods

This meta-analysis was conducted in accordance with the Meta-analysis of Observational Studies in Epidemiology (MOOSE) guidelines and Preferred Reporting Items for Systematic Reviews and Meta-analyses guidelines (PRISMA) [11].

### Search strategy and literature selection

Literature search was performed in the MEDLINE/PubMed, EMBASE and PsychINFO databases by the date of July 1, 2021, using search terms “(((Parkinsonian Disorders) OR (Parkinson disease)) AND (((((genetic variation) OR (gene)) OR (genetic)) OR (inherited)) OR (familial))) AND (((((((((((sleep) OR (sleep wake disorders)) OR (sleep apnea syndromes)) OR (Sleep Initiation and Maintenance Disorders)) OR (Disorders of Excessive Somnolence)) OR (excessive daytime sleepiness)) OR (EDS)) OR (REM Sleep Behavior Disorder)) OR (RBD)) OR (Restless Legs Syndrome)) OR (RLS))”. Studies were selected based on the following inclusion criteria: (1) observational studies, namely cohort, case–control or cross-sectional studies; (2) studies providing specific genotyping methods; and (3) trials incorporating outcomes of sleep disorders by validated tests and comparing the outcomes of PD causative gene variant carriers with those of idiopathic PD (iPD) or HC. The following exclusion criteria were applied: (1) in vitro studies of gene variants; (2) case reports, abstracts for conferences, reviews or meta-analyses; and (3) samples incorporated in multiple cohorts by the same institutions.

To ensure complete retrieval, at preliminary screening we focused on studies related to patients with PD or asymptomatic carriers of gene variants that are considered causative or not. We then narrowed down the selected studies to focus on the relationships between PD pathogenic genes and sleep disorders in PD patients and asymptomatic gene carriers. Quantitative analysis was performed when there were three or more studies on the same gene variant; otherwise, narrative analysis was performed for the same gene variant.

### Risk-of-bias assessments

Two researchers (JH and YC) independently assessed the methodological quality of the included studies using the Newcastle–Ottawa Quality Assessment Scale. Studies with scores > 6 were considered to have a low risk of bias [12].

### Data extraction

Two researchers (JH and YC) independently extracted the following information from each study: the first author, publication year, country, age at assessment, sex, disease duration, disease severity and cognitive status. The sample size, the mean (standard deviation, SD) value of the sleep disturbance test, and the number of sleep disorder events were obtained as primary outcomes. Discrepancies in data extraction were resolved by discussion with a third investigator (CL).

### Statistical analysis

To investigate the relationship between PD causative genes and sleep disorders at the symptomatic and prodromal stages of PD, we divided the studies into two groups: studies on gene variants in patients with PD and studies on gene variants in asymptomatic carriers. In the meta-analysis, we calculated the odds ratio (OR) for binary variables and the standard mean difference (SMD) for continuous variables with 95% confidence intervals (CI) using the Mantel–Haenszel statistical method. Statistical heterogeneity between studies was evaluated used the *I*^2^ test. We adopted the fixed-effect model for pooled analysis when *I*^2^ < 50% and *P* > 0.1, while the random effect model was chosen when *I*^2^ > 50% or *P* < 0.1, considering the relatively high heterogeneity between studies. Subgroup and meta-regression analyses were conducted to explore potential sources of heterogeneity. For publication bias, we adopted Egger’s test and funnel plot asymmetry to evaluate the effect of publication bias when the number of recruited trials was greater than or equal to 10, according to the Cochrane Handbook recommendations [13]. We performed a sensitivity analysis by omitting one study at a time, to ensure the stability of results. All data were analyzed using the STATA (version 16.0) software, and statistical significance was set at *P* < 0.05.

## Results

### Characteristics of the selected studies

**Fig. 1:**
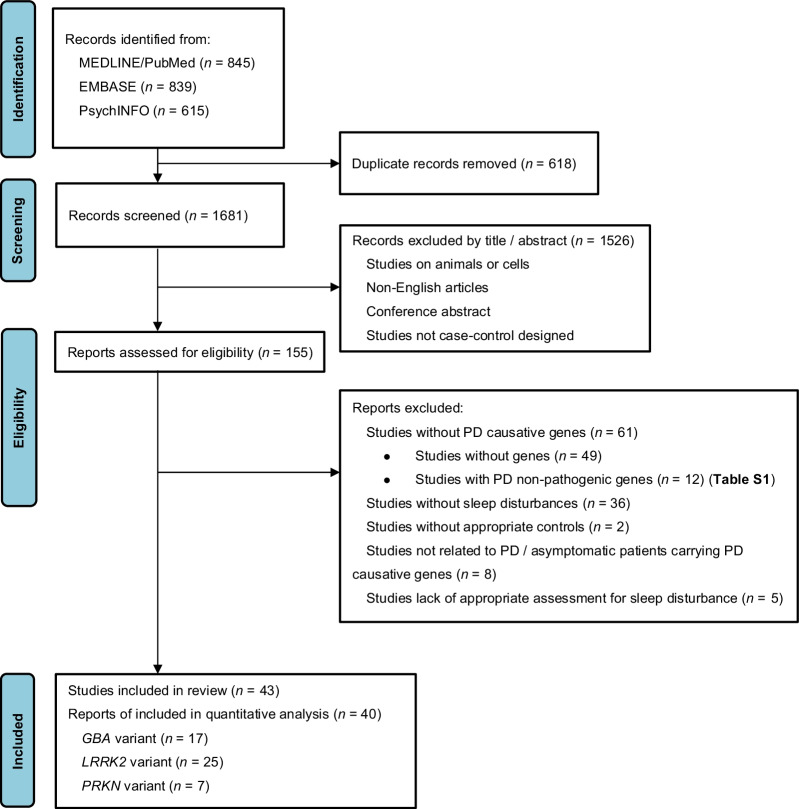
Flowchart of the literature search according to Preferred Reporting Items for Systematic Review and Meta-Analyses (PRISMA). *PD* Parkinson’s disease, *GBA* glucocerebrosidase gene, *LRRK2* Leucine-rich repeat kinase 2 gene, *PRKN* parkin gene

A total of 845 records from MEDLINE (through PubMed), 839 records from EMBASE, and 615 records from PsychINFO were identified at initial search. After removal of duplicates and exclusion by titles and abstracts, 155 articles were retrieved through a full-text review. Moreover, 12 studies regarding non-pathogenic genes of PD in sleep disorders were excluded after further extraction (Additional file 1: Table S1). Finally, 40 studies were selected for quantitative analysis, including studies on *GBA*, *LRRK2* and *PRKN* genes, and three studies on *SNCA* gene were used for qualitative analysis (Fig. 1). Twelve studies were included to compare patients with and without heterozygous *GBA* carriers [5, 14–24], 17 studies to compare patients with and without heterozygous *LRRK2* carriers [15, 16, 20–22, 25–36] and seven studies to compare patients with and without homozygous or compound heterozygous *PRKN* carriers [7, 8, 15, 37–40]. Six studies comparing asymptomatic heterozygous *GBA* carriers with non-carrier HCs were selected [9, 10, 20, 41–43], and 11 studies compared asymptomatic heterozygous *LRRK2* carriers with non-carrier HCs [9, 20, 29–31, 42, 44–48]. The study qualities were evaluated, and one study [18] was considered to have a moderate risk of bias (Additional file 1: Table S2). The general characteristics and outcomes of each study are summarized in Additional file 1: Tables S3 and S4.

### *GBA* variants and the risk of sleep disorders in patients with PD

**Fig. 2:**
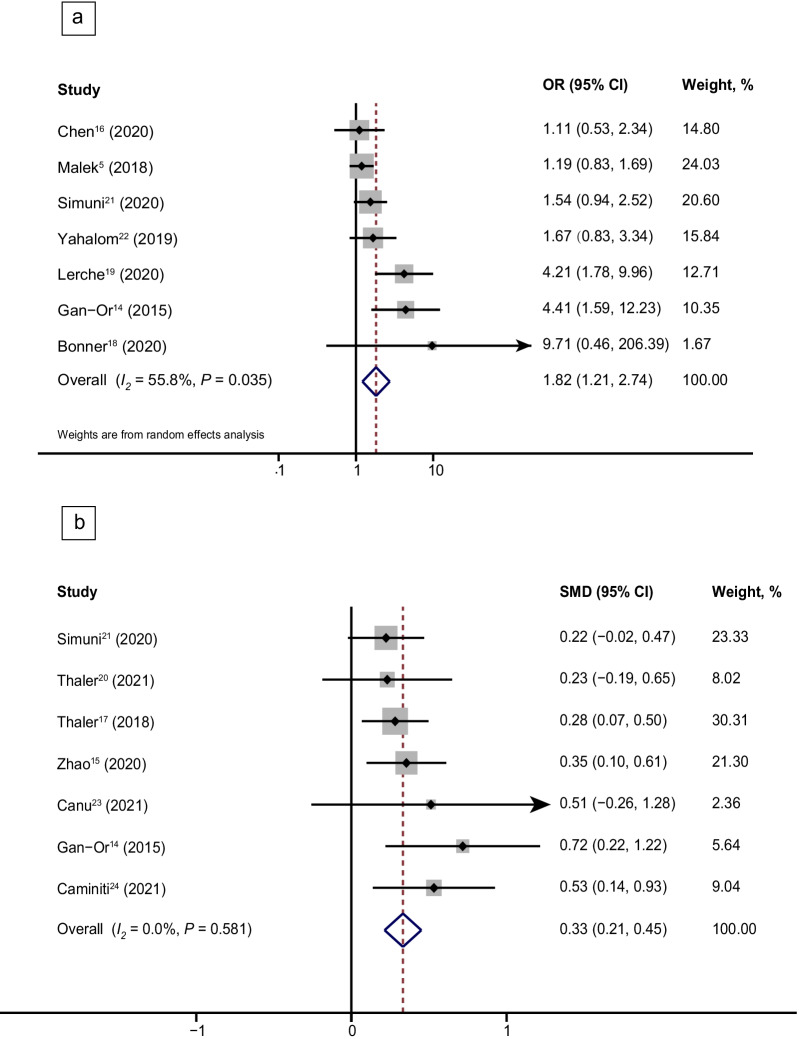
Forest plot of the risk and severity of RBD in PD patients with *GBA* variants. Forest plots display meta-analysis results on the risk (**a**) and severity (**b**) of RBD in PD patients with *GBA* variants compared with iPD. *PD* Parkinson’s disease, *iPD* idiopathic Parkinson’s disease, *GBA* glucocerebrosidase gene, *OR* odds ratio, *SMD* standard mean difference

**Fig. 3:**
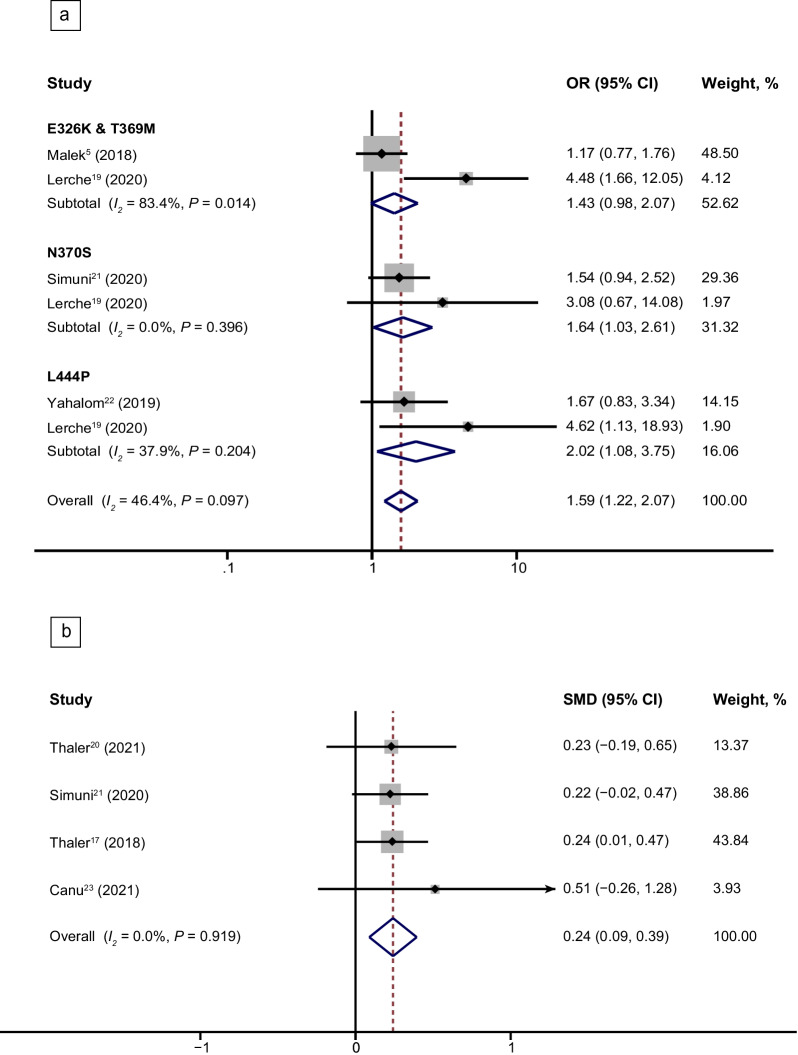
Forest plots of meta-analysis results on the risk (**a**) and severity (**b**) of RBD in PD patient subgroups carrying *GBA* variants compared with iPD. *PD* Parkinson’s disease, *iPD* idiopathic Parkinson’s disease, *GBA* glucocerebrosidase gene, *OR* odds ratio, *SMD* standard mean difference

A total of 12 studies compared sleep disturbance between patients with and without heterozygous *GBA* variants (7 studies on the risk of RBD [5, 14, 16, 18, 19, 21, 22], 7 on the RBD Screening Questionnaire (RBDSQ) score [14, 15, 17, 20, 21, 23, 24] and 2 on the risk of EDS [15, 21]). A random-effect meta-analysis indicated that PD patients carrying heterozygous *GBA* variants had a significantly higher risk of RBD (OR, 1.82; 95% CI, 1.21–2.74, *I*^2^ = 55.8%) (Fig. 2a) and a higher RBDSQ score (SMD, 0.33; 95% CI, 0.21–0.45; *I*^2^ = 0.0%) (Fig. 2b). In the subgroup analysis, the PD patients with N370S [19, 21] and L444P variants [19, 22] of *GBA* were at a higher risk of developing RBD than patients without these variants (N370S: OR, 1.64; 95% CI, 1.03–2.61; *I*^2^ = 0.0; L444P: OR, 2.02; 95% CI, 1.08–3.75; *I*^2^ = 37.9%) (Fig. 3a). Additionally, PD patients carrying the *GBA* N370S variant had higher RBDSQ scores than those without the variant (SMD, 0.24; 95% CI, 0.09–0.39; *I*^2^ = 0.0%) (Fig. 3b) [17, 20, 21, 23]. However, we found that the E326K and T369M variants of *GBA* [5, 19] did not affect the risk of RBD in PD patients (OR, 1.43; 95% CI, 0.98–2.07; *I*^2^ = 83.4%) (Fig. 3a). Sensitivity analysis performed by removing each study demonstrated robustness of the results that *GBA* variants increased the risk and severity of RBD in PD. Nevertheless, no significant difference was found in the severity of EDS between patients with and without *GBA* variants (Additional file 1: Fig. S1). Moderate heterogeneity (*I*^2^ = 55.8%, *P* = 0.035) was found for the risk of RBD in PD patients carrying *GBA* variants. The heterogeneity was diminished when the *GBA* variants were divided into subgroups except for *GBA* E326K and T369M subgroups (*GBA* E326K and T369M, *I*^2^ = 83.4%, *P* = 0.014; *GBA* N370S, *I*^2^ = 0.0%, *P* = 0.396; *GBA* L444P, *I*^2^ = 0.0%, *P* = 0.204). Meta-regression analyses did not identify confounding factors for the outcome of the quantitative analyses (Additional file 1: Table S5). All comparative outcomes of the meta-analysis are displayed in Table 1.

**Table 1:** Summary of comparative outcomes for sleep disturbances

Gene variant	No. of sources	PD with gene variant/controls (*n*)	Main effect		Heterogeneity	
			OR or SMD (95% CI)*	*P* value	*I*^*2*^ statistic (%)	*P* value
RBD in PD patients with/without gene variants						
GBA	7	382/2248	OR: 1.82 (1.21–2.74)	**0.004**	55.8	0.035
E326K&T369M	2	125/1533	OR: 1.43 (0.98–2.07)	0.062	83.4	0.014
N370S	2	86/414	OR: 1.64 (1.03–2.61)	**0.039**	0.0	0.396
L444P	2	70/125	OR: 2.02 (1.08–3.75)	**0.021**	37.9	0.204
GBA	7	478/2149	SMD: 0.33 (0.21–0.45)	**0.000**	0.0	0.581
N370S	4	304/564	SMD: 0.24 (0.09–0.39)	**0.002**	0.0	0.919
*LRRK2*	13	969/3017	OR: 0.69 (0.48–1.00)	0.051	71.7	0.000
G2019S	8	685/1191	OR: 0.49 (0.39–0.61)	**0.000**	0.0	0.639
G2385R	4	266/1450	OR: 1.53 (0.75–3.13)	0.244	75.5	0.007
*LRRK2*	7	512/2858	SMD: − 0.06 (− 0.32 to 0.19)	0.628	78.6	0.000
G2019S	4	347/555	SMD: − 0.33 (− 0.47 to − 0.20)	**0.000**	0.0	0.596
G2385R	2	155 / 759	SMD: 0.21 (− 0.12 to 0.53)	0.218	71.1	0.063
*PRKN*	5	122/337	OR: 0.74 (0.29–1.86)	0.519	60.9	0.037
*PRKN*	2	107/1568	SMD: − 0.17 (− 0.37 to 0.04)	0.109	49.1	0.161
RBD in asymptomatic patients with gene variants and HC						
*GBA*	4	261/370	OR: 1.04 (0.68–1.59)	0.861	0.0	0.920
*GBA*	2	135/81	OR: 0.22 (− 0.08 to 0.52)	0.158	0.0	0.333
*GBA* (baseline)	2	43/38	SMD: − 0.26 (− 0.69 to 0.18)	0.252	0.0	0.922
GBA (follow-up)	2	43/38	SMD: 0.63 (0.18–1.08)	**0.006**	0.0	0.874
*LRRK2*	6	615/610	OR: 0.89 (0.52–1.52)	0.659	53.0	0.059
G2019S	5	551/562	OR: 0.81 (0.59–1.11)	0.195	44.6	0.124
*LRRK2*	6	457/389	SMD: 0.08 (− 0.25 to 0.41)	0.636	79.3	0.000
EDS in PD patients with/without gene variants						
*GBA*	2	136/1786	OR: 0.02 (− 0.16 to 0.20)	0.832	0.0	0.437
*LRRK2*	5	289/1269	OR: 1.29 (0.59–2.82)	0.530	65.4	0.021
G2019S	3	111/89	OR: 2.49 (0.92–6.72)	0.073	37.2	0.204
G2385R	2	178/1180	OR: 0.79 (0.57–1.09)	0.152	0.0	0.427
*LRRK2*	7	379/2628	SMD: 0.05 (− 0.07 to 0.17)	0.457	19.0	0.284
G2019S	5	290/550	SMD: 0.05 (− 0.10 to 0.19)		0.0	0.689
*PRKN*	2	30/91	OR: 0.21 (0.04–1.23)	0.084	0.0	0.424
*PRKN*	4	171/1787	SMD: 0.11 (− 0.3 to 0.53)	0.601	75.9	0.006
EDS in asymptomatic patients with gene variants and HC						
*LRRK2*	4	398/320	OR: 1.06 (0.68–1.65)	0.811	0.0	0.446
*LRRK2*	4	251/227	SMD: − 0.10 (− 0.43 to 0.24)	0.574	57.8	0.068
RLS in PD patients with/without gene variants						
*LRRK2*	3	276/1199	OR: 0.81 (0.60–1.11)	0.186	0.0	0.918
G2019S	2	113/137	OR: 0.78 (0.36–1.69)	0.525	0.0	0.692
*PRKN*	3	41/102	OR: 1.01 (0.08–13.22)	0.995	74.6	0.020
PDSS score in PD patients with/without gene variants						
*LRRK2*	3	67/170	SMD: 0.12 (− 0.20 to 0.43)	0.463	16.7	0.301
G2019S	2	51/52	SMD: 0.19 (− 0.20 to 0.58)	0.336	50.2	0.156

Bold type indicates statistical significant outcomes

*OR* odds ratio, *SMD* standard mean difference, *RBD* rapid eye movement (REM) sleep behavior disorder, *EDS* excessive daytime sleepiness, *RLS* restless legs syndrome, *PDSS* Parkinson Disease Sleep Scale, *GBA* glucocerebrosidase gene, *LRRK2* Leucine-rich repeat kinase 2 gene, *PRKN* parkin gene, *HC* healthy control

^a^OR indicates odds ratio for the risk of sleep disturbances; SMD indicates standard mean difference for the severity of sleep disturbances evaluated by scales such as RBD Screening Questionnaire, Epworth Sleepiness Scale and Parkinson Disease Sleep Scale

### *LRRK2* variants and the risk of sleep disorders in patients with PD

**Fig. 4:**
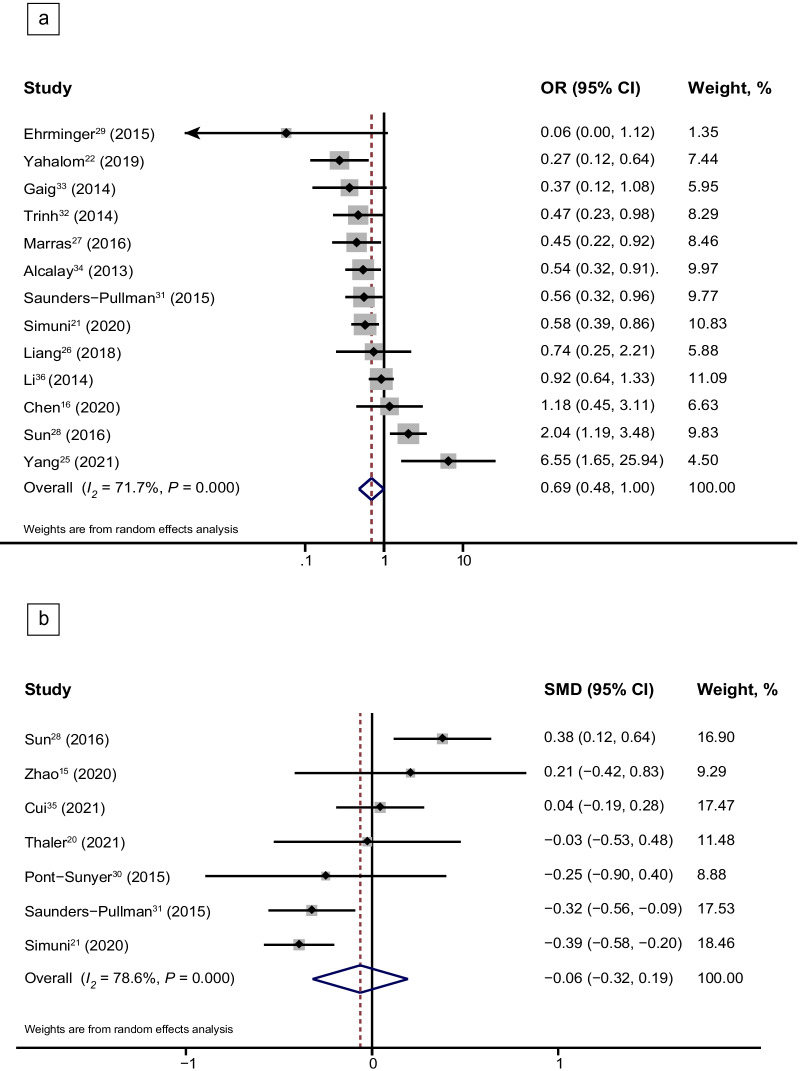
Forest plots displaying meta-analysis results on the risk (**a**) and severity (**b**) of RBD in PD patients with *LRRK2* variants compared with iPD. *PD* Parkinson’s disease, *iPD* idiopathic Parkinson’s disease, *LRRK2* Leucine-rich repeat kinase 2 gene, *OR* odds ratio, *SMD* standard mean difference

**Fig. 5:**
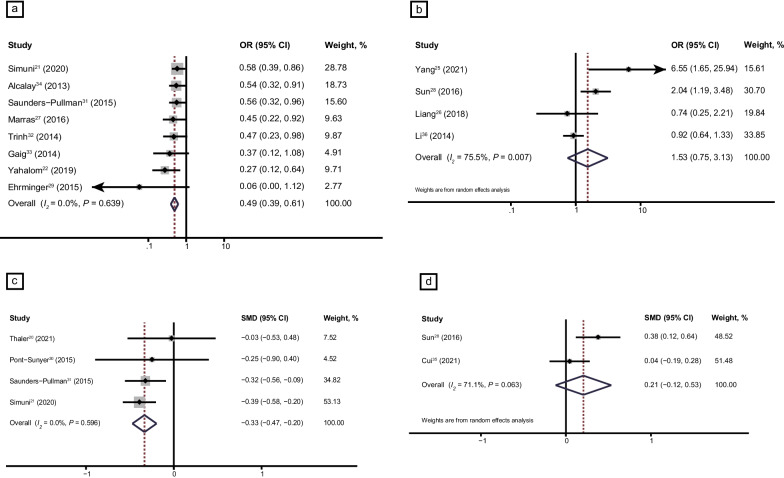
Forest plots displaying meta-analysis results on the risk of RBD in PD patients with *LRRK2* G2019S variant (**a**) and *LRRK2* G2385R variant (**b**) compared with iPD, as well as severity of RBD in PD patients with *LRRK2* G2019S variant (**c**) and *LRRK2* G2385R variant (**d**) compared with iPD. *PD* Parkinson’s disease, *iPD* idiopathic Parkinson’s disease, *LRRK2* Leucine-rich repeat kinase 2 gene, *OR* odds ratio, *SMD* standard mean difference

Pooled analysis of 13 studies [16, 21, 22, 25–29, 31–34, 36] showed that there was no significant difference in the risk (OR, 0.69; 95% CI, 0.48–1.00; *I*^2^ = 71.7%) of RBD between patients with and without LRRK2 variants. There was also no significant difference in the severity of RBD (SMD, −0.06; 95% CI, − 0.32 to 0.19; *I*^2^ = 78.6%) from seven studies [15, 20, 21, 28, 30, 31, 35] between patients with and without *LRRK2* variants (Fig. 4a, b). High heterogeneity was found in the studies on the prevalence and severity of RBD. Subgroup analysis of *LRRK2* G2019S [21, 22, 27, 29, 31–34] and *LRRK2* G2385R variants [25, 26, 28, 36] indicated that PD patients carrying *LRRK2* G2019S had 51% lower odds of the risk of RBD compared to those without *LRRK2* G2019S (OR, 0.49; 95% CI, 0.39–0.61; *I*^2^ = 0.0%) (Fig. 5a), whereas *LRRK2* G2385R had no effect on the risk of RBD (OR, 1.53; 95% CI, 0.75–3.13; *I*^2^ = 75.5%) (Fig. 5b). PD patients carrying the *LRRK2* G2019S variant [20, 21, 30, 31] had lower RBDSQ scores than those without *LRRK2* G2019S (SMD, − 0.33; 95% CI, − 0.47 to − 0.20; *I*^2^ = 0.0%) (Fig. 5c), while there was no difference in RBDSQ score between patients with and without *LRRK2* G2385R [28, 35] (Fig. 5d). There was no risk of EDS in patients with *LRRK2* variants (Additional file 1: Fig. S2a, S2b), or in the subgroup of patients with the *LRRK2* G2019S variant (Additional file 1: Fig. S2c). There were no differences in the Parkinson Disease Sleep Scale score or the risk of RLS between patients with and without *LRRK2* variants (Additional file 1: Fig. S3a, S3b). Sensitivity analysis demonstrated that the pooled ORs and SMDs of RBD in patients with and without *LRRK2* variants were stable. Egger’s test showed no publication bias (*P* = 0.672). Funnel plot of studies on RBD risk in PD patients carrying *LRRK2* G2019S is shown in Additional file 1: Fig. S4. High heterogeneity was discovered in the risk and severity of RBD in patients with PD carrying *LRRK2* variants (risk of RBD, *I*^2^ = 71.7%, *P* = 0.000; severity of RBD, *I*^2^ = 78.6%, *P* = 0.000). Subgroup analysis revealed that there was no heterogeneity in *LRRK2* G2019S variants (risk of RBD, *I*^2^ = 0.0%, *P* = 0.639; severity of RBD, *I*^2^ = 0.0%, *P* = 0.596) while high heterogeneity still existed in *LRRK2* G2385R variant among studies (risk of RBD, *I*^2^ = 75.5%, *P* = 0.007; severity of RBD, *I*^2^ = 71.1%, *P* = 0.063). Age, sex, disease duration, disease stage and cognitive status were not confounding factors for the results of meta-regression of *LRRK2* G2385R groups (Additional file 1: Table S5).

### *PRKN* variants and risk of sleep disorders in patients with PD

We found no significant difference in the risk of RBD (OR, 0.74; 95% CI, 0.29–1.86; *I*^2^ = 60.9%), EDS (OR, 0.21; 95% CI, 0.04–1.23; *I*^2^ = 0.0%), or RLS (OR, 1.01; 95% CI, 0.08–13.22; *I*^2^ = 74.6%) between PD patients carrying homozygous or compound heterozygous *PRKN* variants and iPD (Additional file 1: Figs. S5a, S6a, S7). *PRKN* variants did not worsen or alleviate the severity of RBD or EDS (Additional file 1: Figs. S5b, S6b).

### Genetic heterogeneity and risk of sleep disorders in asymptomatic carriers

Pooled analysis of cross-sectional studies demonstrated that there were no significant differences in the risk of RBD (OR, 1.04; 95% CI, 0.68–1.59; *I*^2^ = 0.0%) [9, 10, 41, 42] (Additional file 1: Fig. S8a) and RBDSQ score (SMD, 0.22; 95% CI, −0.08 to 0.52; *I*^2^ = 0.0%) [20, 41] between asymptomatic heterozygous *GBA* carriers and non-carrier HCs (Additional file 1: Fig. S8b). However, quantitative analysis of longitudinal cohort studies demonstrated an increased RBDSQ score over follow-up (SMD, 0.63; 95% CI, 0.18–1.08; *I*^2^ = 0.0%) in asymptomatic heterozygous *GBA* carriers than in the non-carrier HCs (Additional file 1: Fig. S8d), despite no difference at baseline between the two groups [10, 43] (SMD, −0.26; 95% CI, −0.69 to 0.18; *I*^2^ = 0.0%) (Additional file 1: Fig. S8c). *LRRK*2 variant did not affect the risk and severity of RBD or EDS in asymptomatic *LRRK*2 carriers (Additional file 1: Fig. S9 and S10).

### Abnormal sleep architecture in PD patients and asymptomatic carriers of *SNCA* variant

Three small-sized studies investigated the association between p.A53T (p.Ala53Thr, c.209G > A) variant of *SNCA* and sleep disturbance in PD patients or asymptomatic carriers. Simitsi et al. [49] recruited 15 PD patients carrying the *SNCA* p.A53T variant and found a higher prevalence of RBD in these patients than in iPD. However, Koros et al. [50] did not find a difference in sleep disturbances including insomnia, RBD and EDS between patients with and without *SNCA* p.A53T. Regarding the prodromal stages of PD, two studies found a low prevalence of RBD in *SNCA* p.A53T asymptomatic carriers [49, 51].

## Discussion

In this systematic review and meta-analysis, we examined the associations between variants of causative genes of PD and sleep disturbance in both PD patients and asymptomatic carriers at the prodromal phase of PD. Our findings suggest that variants of the causative genes play a role in sleep disturbances in PD. We found that *GBA* variants increased the risk and severity of RBD in patients with PD, while the *LRRK2* G2019S variant reduced the risk and severity of RBD. In addition, *GBA* variants worsened the RBD symptoms at the prodromal stage of PD, while variants of *GBA* and *LRRK2* did not influence the risk of EDS in patients with PD. *PRKN* variants did not influence the risk of RBD, EDS, or RLS in PD.

Our study revealed relationships between *GBA* variants and RBD in patients with PD. *GBA* encodes beta-glucocerebrosidase, and biallelic pathogenic variants of *GBA1* cause Gaucher disease (GD), a lysosomal disorder. Both homozygous and heterozygous *GBA* variants increase the risk of PD [52]. *GBA* variants are classified into severe variants (such as L444P, W291X, H225Q, and IVS2 + 1G > A) and mild variants (such as N370S). In addition, several non-pathogenic variants of *GBA* in GD, such as E326K and T369M, have been found to increase the risk of PD [53]. PD patients with severe variants of *GBA* have a younger age of onset, faster progression and more severe cognitive impairment than those with mild variants [54]. In this meta-analysis, we divided *GBA* variants into groups based on the severity of *GBA* variants (L444P regarded as the severe variant, N370S as the mild variant, and E326K & T369M as the non-pathogenic variants), and showed that the pathogenic variants of *GBA* increase the risk of RBD. Notably, the OR of *GBA* L444P variant (2.02) [19, 22] for RBD in PD patients was higher than that of the *GBA* N370S variant (OR 1.68) [19, 21]. However, there were no differences in the risk of RBD between patients with and without *GBA* E326K or T369M variant [5, 19]. Our current findings indicate that the role of *GBA* variants in increasing the risk of RBD in PD differs depending on the severity of *GBA* variants. RBD is considered as a prodrome of α-synucleinopathy, and *GBA* gene is closely correlated with α-synuclein. Impaired proteolysis caused by GCase deficiency preferentially increases α-synuclein deposition and spread of α-synuclein pathology, while elevated α-synuclein decreases the GCase activity [55]. Lower levels of α-synuclein in CSF have been detected in PD patients carrying *GBA* variants compared to iPD patients, with a downward trend depending on the order of severe, mild and risk variants [19]. Differences in α-synuclein expression in different *GBA* variants may elucidate the discrepancy in phenotypes.

For the prodromal stage of PD, our meta-analysis found that *GBA* variant carriers had worse RBD symptoms during the follow-up period [10, 43]. Interestingly, no difference was found in the severity of RBD between *GBA* variant carriers and HCs at baseline [10, 43]. Statistical analysis of cross-sectional studies failed to find any difference in risk [9, 10, 41, 42] or severity [20, 41] between asymptomatic carriers of *GBA* variants and HCs. These findings suggest that *GBA* variant is a risk factor for the development of RBD at the prodromal stage with disease duration.

*LRRK2* is one of the causative genes for autosomal dominant PD. Previous cross-sectional studies have indicated that patients with PD carrying the *LRRK2* G2019S variant, the most common variant, exhibit slower disease progression and milder motor symptoms than iPD patients [56]. Our meta-analysis found that *LRRK2* G2019S decreased the risk and severity of RBD [21, 22, 27, 29–34], but *LRRK2* G2385R did not affect the risk of RBD in patients with PD [25, 26, 28, 36]. In prodromal stages, our pooled analysis suggested that *LRRK2* G2019S did not increase or decrease the risk and severity of RBD in asymptomatic carriers, consistent with a review by Tolosa et al. [57]. In addition, our meta-analysis found no associations between other sleep disturbances, including EDS and RLS, and *LRRK2* G2019S and G2385R variants. Further studies are needed to elucidate the associations between EDS/RLS and *LRRK2* variants.

The pathophysiological mechanisms of *LRRK2* variants in PD are related to a variety of pathways such as kinase activity, autophagy and oxidative stress. The interplay between α-synuclein and *LRRK2* mutations accelerates the progression of α-synuclein-mediated neurodegeneration, with Rab proteins and chaperones serving as mediators [58]. However, only half of PD patients carryin*g LRRK2* variants were found to contain brainstem synucleinopathy in postmortem studies, suggesting that some *LRRK2* variants may not be involved in central synucleinopathy [59]. Most PD patients with *LRRK2* variants display loss of dopaminergic neurons in the substantia nigra. Interestingly, patients carrying the *LRRK2* G2019S variant show more Lewy body pathology than patients with other *LRRK2* variants [60]. Skin biopsy revealed no difference in deposition of phosphorylated α-synuclein between *LRRK2* G2385R carriers and non-carriers [25], whereas high levels of α-synuclein deposition in sympathetic noradrenergic nerves in skin biopsies were found in patients with *LRRK2* G2019S and R1441G variants [59]. Reasons for the low risk of RBD in PD patients carrying the *LRRK2* G2019S variant require more pathological research for interpretation.

*PRKN* is one of the most frequently mutated genes in patients with early-onset PD [15] and is involved in the mitochondrial function of neural cells. Hatice Kumru found that six out of 10 PD patients carrying *PRKN* variants (7 carrying homozygous *PRKN* variants) developed mild RBD as assessed by video polysomnography [61]. However, our pooled analysis of cross-sectional studies showed no correlation between *PRKN* variant and the risk and severity of sleep disturbances including RBD [7, 8, 15, 37–40], EDS [7, 8, 15, 37, 38, 40] and RLS [7, 8, 40] in patients with PD. At the prodromal stage of PD, only one study showed no difference in the risk of RBD between asymptomatic carriers with and without *PRKN* variants [62]. Large-scale studies are urgently required to dispel the mist of inconsistent results on sleep disorders in patients with *PRKN* variants.

*SNCA* encodes α-synuclein proteins, which abnormally accumulate in PD as a major component of Lewy bodies. Observational studies in cases and families have reported RBD in PD patients carrying various variants of *SNCA*, such as A53T variant, duplications [63] and triplications [64]. However, two studies on the risk of RBD in PD patients carrying *SNCA* A53T [49, 50] demonstrated contradictory results. As for the prodromal stage of PD, carriers of *SNCA* A53T reported RBD before the appearance of motor symptoms in a longitudinal study [51]. Moreover, *SNCA* methylation may play a role in the process of *SNCA* expression, which is associated with RBD. Previous studies have found hypomethylation in the promoter and intron 1 regions of *SNCA* in idiopathic RBD patients and PD patients compared to HCs [65].

Patients carrying *GBA*, *LRRK2*, *PRKN*, or *SNCA* variants have different manifestations and pathogeneses in essence. Patients with *SNCA*, *LRRK2*, or *GBA* variants have peripheral synucleinopathy, while patients with *PRKN* variants do not [59]. Moreover, PD patients with *PRKN* variants mostly lack α-synuclein deposition in the brain and part of PD patients with *LRRK2* variants have α-synuclein deposition in the central nervous system. *GBA*, *LRRK2* and *SNCA* variants are associated with lysosomal dysfunction, while the *PRKN* variant is directly involved in mitochondrial functions [66]. It has been proposed that patients with *GBA* or *SNCA* variants typically have initial pathological α-synuclein originating in the enteric and autonomic nervous systems, whereas patients with *LRRK2* or *PRKN* variants have initial α-synuclein pathology originating in the brain [67].

## Limitations

This systematic review and meta-analysis had some limitations. First, the studies included were associated with various disease durations and ages of patients at enrollment. Due to the limited number of studies, we did not perform a pooled analysis stratified by disease duration or age, even though the meta-regression showed that age, sex, disease duration, disease stage and cognitive status did not contribute to the heterogeneity of enrolled studies. Second, the included studies were conducted in different countries, indicating different genetic characteristics due to different regions. It is worth mentioning that studies on *LRRK2* G2385R were from Asian regions since this risk variant of PD is more common in individuals of Asian descent. In contrast, the *LRRK2* G2019S variant is rare in Asian populations. Third, the limited case–control studies of *SNCA* variants restricted us from performing quantitative analysis, even though *SNCA* is a causative gene for PD and directly correlates to pathological hallmark of PD. Moreover, other causative genes of PD, such as *PINK1* and *DJ-1*, were not discussed in this meta-analysis due to the lack of original studies. Fourth, most diagnoses of sleep disturbances in the included studies were made through clinical evaluation scales but not polysomnography; therefore, more accurate techniques such as polysomnography should be used. Moreover, the studies included in the meta-analysis were mostly cross-sectional. A lack of prospective longitudinal study with a larger number of patients may result in a failure to discover the difference in sleep disturbance between cases and controls, especially between genetic asymptomatic carriers and non-carrier controls.

## Conclusions

In conclusion, this meta-analysis showed that some PD causative genes are associated with sleep disorders in PD patients at clinical stages, as well as at the asymptomatic stage. *GBA* variants can increase the risk and severity of RBD in the clinical stages of PD and also increase the severity of RBD in the prodromal stage of PD, while *LRRK2* G2019S is negatively associated with RBD in patients with PD. These findings provide evidence that genetic heterogeneities play a role in the development of sleep disturbance, especially RBD, in PD patients and at the prodromal stage of PD. These genetic heterogeneities indicate involvement of several common pathways and pathogenesis between PD and sleep disorders, such as α-synuclein aggregation in both PD and RBD. Longitudinal studies and investigations on more PD causative or risk genes are needed to explore the relations between PD and sleep disorders, leading to precise recognition and target therapy for PD patients and gene carriers prone to sleep disorders, such as RBD in patients and carriers of *GBA* variants.

## Supplementary Information

**Additional file 1: Table S1. **Studies excluded because of non-pathogenic genes of PD**. Table S2.** Study Evaluation according to Newcastle-Ottawa Quality Assessment Scale. **Table S3.** General characteristics of included studies. **Table S4.** Primary outcomes of included studies. **Table S5.** Results of meta-regression in patients with gene variants. **Fig. S1.** Severity of EDS in PD patients with and without GBA variants. **Fig. S2.** Risk (a) and severity (b) of EDS in PD patients with and without LRRK2 variants, severity of EDS in PD patients with and without LRRK2 G2019S variants (c). **Fig. S3.** PDSS score (a) and risk of RLS (b) in PD patients with and without LRRK2 variants. **Fig. S4.** Funnel plots for the risk of RBD in PD patients with LRRK2 variants. **Fig. S5.** Risk (a) and severity (b) of RBD in PD patients with PRKN variants. **Fig. S6.** Risk (a) and severity (b) of EDS in PD patients with PRKN variants. **Fig. S7.** Risk of RLS in PD patients with and without PRKN variants. **Fig. S8.** Risk (a) and severity (b) of RBD in asymptomatic carriers with GBA variant and HCs. **Fig. S9.** Risk (a) and severity (b) of RBD in asymptomatic carriers with LRRK2 G2019S and HCs. **Fig. S10.** Risk (a) and severity (b) of EDS in asymptomatic carriers with LRRK2 G2019S and HCs.

## Data Availability

All data generated or analyzed during this study are included in Additional file 1.

## Abbreviations

PD: Parkinson’s disease
EDS: Excessive daytime sleepiness
RLS: Restless legs syndrome
RBD: Rapid-eye-movement behavior disorders
*SNCA*: Alpha-synuclein gene
*LRRK2*: Leucine-rich repeat kinase 2 gene
*GBA*: Glucocerebrosidase gene
*PRKN*: Parkin gene
HC: Healthy controls
OR: Odds ratios
SMD: Standard mean difference
CI: Confidence intervals
RBDSQ: RBD Screening Questionnaire
