# Supplementary material for: Genetic heterogeneity on sleep disorders in Parkinson’s disease: a systematic review and meta-analysis
Source: Transl Neurodegener. 2022 Apr 8;11:21. doi: 10.1186/s40035-022-00294-1 (PMC8991652; doi:10.1186/s40035-022-00294-1)
Supplement: Supplementary file 1 — Additional file 1: Table S1. Studies excluded because of non-pathogenic genes of PD. Table S2. Study Evaluation according to Newcastle-Ottawa Quality Assessment Scale. Table S3. General characteristics of included studies. Table S4. Primary outcomes of included studies. Table S5. Results of meta-regression in patients with gene variants. Fig. S1. Severity of EDS in PD patients with and without GBA variants. Fig. S2. Risk (a) and severity (b) of EDS in PD patients with and without LRRK2 variants, severity of EDS in PD patients with and without LRRK2 G2019S variants (c). Fig. S3. PDSS score (a) and risk of RLS (b) in PD patients with and without LRRK2 variants. Fig. S4. Funnel plots for the risk of RBD in PD patients with LRRK2 variants. Fig. S5. Risk (a) and severity (b) of RBD in PD patients with PRKN variants. Fig. S6. Risk (a) and severity (b) of EDS in PD patients with PRKN variants. Fig. S7. Risk of RLS in PD patients with and without PRKN variants. Fig. S8. Risk (a) and severity (b) of RBD in asymptomatic carriers with GBA variant and HCs. Fig. S9. Risk (a) and severity (b) of RBD in asymptomatic carriers with LRRK2 G2019S and HCs. Fig. S10. Risk (a) and severity (b) of EDS in asymptomatic carriers with LRRK2 G2019S and HCs. [file 40035_2022_294_MOESM1_ESM.docx]

**Table S1 Studies excluded because of non-pathogenic genes of PD**

| **Author (year)** | **Gene** | **Sleep disorders** |
| --- | --- | --- |
| Hua (2021)(1) | TEF | PDSS |
| Lin (2019)(2) | ALDH2 | EDS, RBD, PDSS |
| Li (2018)(3) | MAPT | RBD |
| Lou (2018)(4) | CLOCK | PSQI |
| Mohtashami (2018)(5) | TOX3 | RLS |
| Gao (2010)(6) | BDNF | RBD, OSAS |
| Rissling (2006)(7) | COMT | EDS |
| Frauscher (2004)(8) | COMT | EDS |
| Zhang (2020)(9) | ZNF184 | RBD, EDS |
| Kim (2021)(10) | APOE | RBD, EDS |
| Deutschlander (2020)(11) | MAPT | RBD, RLS |
| Wu (2020)(12) | NMD3 | RBD, RLS, EDS |

Abbreviations: TEF, thyrotroph embryonic factor gene; ALDH2, aldehyde dehydrogenase 2; BDNF, brain-derived neurotrophic factor; COMT, catechol-O-methyltransferase; APOE, apolipoprotein E; RBD, rapid eye movement (REM) sleep behavior disorder; EDS, excessive daytime sleepiness; RLS, restless legs syndrome; PDSS, Parkinson Disease Sleep Scale; PSQI, Pittsburgh Sleep Quality Index; OSAS, obstructive sleep apnea syndrome

**Table** **S2 Study Evaluation according to Newcastle-Ottawa Quality Assessment Scale**

| **Source** | **Study Design** | **Selection** | **Comparability** | **Exposure/Outcome** | **Total Score** |
| --- | --- | --- | --- | --- | --- |
| Chen *et al* (2020) | cross-sectional | 4 | 2 | 1 | 7 |
| Malek *et al* (2018) | cross-sectional | 4 | 2 | 1 | 7 |
| Simuni *et al* (2020) | cross-sectional | 4 | 2 | 3 | 9 |
| Yahalom *et al* (2019) | cross-sectional | 4 | 2 | 1 | 7 |
| Lerche *et al* (2020) | cross-sectional | 4 | 2 | 1 | 7 |
| Gan-Or *et al* (2015) | cross-sectional | 3 | 2 | 2 | 7 |
| Bonner *et al* (2020) | cross-sectional | 2 | 2 | 1 | 5 |
| Thaler *et al* (2021) | cross-sectional | 4 | 1 | 1 | 6 |
| Thaler *et al* (2018) | cross-sectional | 4 | 1 | 1 | 6 |
| Zhao *et al* (2020) | cross-sectional | 4 | 2 | 1 | 7 |
| Canu *et al* (2021) | cross-sectional | 4 | 1 | 2 | 7 |
| Caminiti et al (2021) | cross-sectional | 4 | 1 | 1 | 6 |
| Moran *et al* (2021) | cross-sectional | 4 | 1 | 1 | 6 |
| Simuni *et al* (2020) | cross-sectional | 4 | 2 | 2 | 8 |
| Chahine *et al* (2018) | cross-sectional | 4 | 2 | 1 | 7 |
| Ehrminger *et al* (2015) | cross-sectional | 4 | 2 | 1 | 7 |
| Gaig *et al* (2014) | cross-sectional | 4 | 2 | 1 | 7 |
| Trinh *et al* (2014) | cross-sectional | 4 | 2 | 1 | 7 |
| Marras *et, al* (2016) | cross-sectional | 4 | 2 | 1 | 7 |
| Alcalay *et al* (2013) | cross-sectional | 4 | 1 | 1 | 6 |
| Saunders-Pullman *et al* (2015) | cross-sectional | 4 | 2 | 1 | 7 |
| Liang *et al* (2018) | cross-sectional | 3 | 2 | 2 | 7 |
| Li *et al* (2015) | cross-sectional | 4 | 2 | 1 | 7 |
| Sun *et al* (2016) | cross-sectional | 4 | 2 | 1 | 7 |
| Yang *et al* (2021) | cross-sectional | 4 | 2 | 1 | 7 |
| Cui *et al* (2021) | cross-sectional | 4 | 2 | 1 | 7 |
| Pont-Sunyer *et al* (2015) | cross-sectional | 4 | 2 | 2 | 8 |
| Johansen *et al* (2011) | cross-sectional | 3 | 1 | 2 | 6 |
| Pont-Sunyer *et al* (2017) | cross-sectional | 4 | 1 | 3 | 8 |
| van den Heuve *et al* (2018) | cross-sectional | 3 | 2 | 1 | 6 |
| Mirelman *et al* (2015), Israel | cross-sectional | 3 | 2 | 1 | 6 |
| Mestre *et al* (2018) | cross-sectional | 4 | 2 | 2 | 8 |
| Morgante *et al* (2016) | cross-sectional | 4 | 2 | 1 | 7 |
| Song *et al* (2020) | cross-sectional | 4 | 0 | 2 | 6 |
| Kim *et al* (2011) | cross-sectional | 4 | 1 | 1 | 6 |
| Kagi *et al* (2010) | cross-sectional | 3 | 1 | 2 | 6 |
| Limousin *et al* (2009) | cross-sectional | 4 | 1 | 2 | 7 |
| Zhou *et al* (2020) | cross-sectional | 4 | 1 | 2 | 7 |
| Avenali *et al* (2019) | cohort | 3 | 1 | 2 | 6 |
| Beavan *et al* (2015) | cohort | 3 | 1 | 2 | 6 |

**Table S3 General characteristics of included studies**

| **Source (y), country** | **Gene variants** | **Sample size (n)** | **Sex (male, %)** | **Age at enrollment, mean (SD), y** | **Disease duration, mean (SD), y** | **UPDRS III, mean (SD)** | **MoCA, mean (SD)** |
| --- | --- | --- | --- | --- | --- | --- | --- |
|  |  |  |  |  |  |  |  |
| Chen *et al* (2020), China | *GBA*, *LRRK2* | *GBA*-PD: 32  *LRRK2*-PD: 18  iPD: 376 | *GBA*-PD: 41.7  *LRRK2*-PD: 35.0  iPD: 55.7 | *GBA*-PD: 46 (9.50)  *LRRK2*-PD: 47 (6.41)  iPD: 44.9 (7.94) | *GBA*-PD: 5.1 (8.2)  *LRRK2*-PD: 5.6 (5.5)  iPD: 5.6 (5.2) | *GBA*-PD: 12.3 (10.9)  *LRRK2*-PD: 10.7 (9.8)  iPD: 9.2 (8.2) | *GBA*-PD: 24.9 (3.9)  *LRRK2*-PD: 23.1 (3.7)  iPD: 24.9 (4.3) |
| Malek *et al* (2018), UK | *GBA* | *GBA*-PD group 1^*^: 44  *GBA*-PD group 2^**^: 99  iPD: 1584 | *GBA*-PD group 1: 63.6  *GBA*-PD group 2: 66.3  iPD: 65.4 | *GBA*-PD group 1: 62.9 (12.3)  *GBA*-PD group 2: 66.8 (8.7)  iPD: 67.6 (9.2) | *GBA*-PD group 1: 1.5 (1.1)  *GBA*-PD group 2: 1.2 (0.9)  iPD: 1.3 (0.9) | *GBA*-PD group 1: 24.2 (13.0)  *GBA*-PD group 2: 21.8 (11.5)  iPD: 22.6 (12.2) | *GBA*-PD group 1: 25.9 (2.7)  *GBA*-PD group 2: 25.0 (3.3)  iPD: 25.2 (3.5) |
| Simuni *et al* (2020), USA | *GBA, LRRK2* | *GBA*-PD*:* 80  *LRRK2*-PD: 158  iPD: 361 | *GBA*-PD: 53.8  *LRRK2*-PD: 48.1  iPD: 65.9 | *GBA*-PD: 62.7 (9.9)  *LRRK2*-PD: 63.8 (9.2)  iPD: 63.8 (9.7) | *GBA*-PD: 3.1 (2.0)  *LRRK2*-PD: 2.9 (1.9)  iPD: 2.6 (0.6) | *GBA*-PD†: 26.2 (10.8)  *LRRK2*-PD†: 22.1 (11.6)  iPD†: 27.2 (11.1) | *GBA*-PD: 26.1 (2.9)  *LRRK2*-PD: 25.9 (3.2)  iPD: 26.2 (3.2) |
| Yahalom *et al* (2019), Israel | *GBA*  *LRRK2* | *GBA*-PD*:* 78  *LRRK2*-PD: 66  iPD: 80 | *GBA*-PD: 60.3  *LRRK2*-PD: 57.6  iPD: 65 | *GBA*-PD: 70.1 (12.6)  *LRRK2*-PD: 72.1 (13.6)  iPD: 72.7 (13.4) | *GBA*-PD: 11.5 (7.0)  *LRRK2*-PD: 14.4 (8.3)  iPD: 11.3 (6.5) | *GBA*-PD: 37.3 (18.1)  *LRRK2*-PD: 30.9 (19.1)  iPD: 29.6 (11.2) | NA |
| Lerche *et al* (2020), Germany | *GBA* | *GBA*-PD group 1^#^: 21  *GBA*-PD group 2^##^: 16  *GBA*-PD group 3^###^: 43  iPD: 80 | *GBA*-PD group 1: 71  *GBA*-PD group 2: 69  *GBA*-PD group 3: 67  iPD: 70 | *GBA*-PD group 1: 59 (10)  *GBA*-PD group 2: 65 (9)  *GBA*-PD group 3: 66 (10)  iPD: 64 (10) | *GBA*-PD group 1: 8 (6)  *GBA*-PD group 2: 8 (4)  *GBA*-PD group 3: 8 (5)  iPD: 7 (4) | *GBA*-PD group 1: 29 (10)  *GBA*-PD group 2: 39 (16)  *GBA*-PD group 3: 28 (11)  iPD: 28 (12) | *GBA*-PD group 1: 23 (5)  *GBA*-PD group 2: 24 (6)  *GBA*-PD group 3: 24 (5)  iPD: 26 (4) |
| Gan-Or *et al* (2015), Canada | *GBA* | *GBA*-PD: 19  iPD: 101 | NA | NA | NA | NA | NA |
| Bonner *et al* (2020), UK | *GBA* | *GBA*-PD: 15  iPD: 5 | *GBA*-PD: 53  iPD: 40 | *GBA*-PD: 62.3 (13.6)  iPD: 64.9 (9.8) | NA | NA | NA |

**Table S3 continued**

| **Source (y), country** | **Gene variants** | **Sample size (n)** | **Sex (male, %)** | **Age at enrollment, mean (SD), y** | **Disease duration, mean (SD), y** | **UPDRS III, mean (SD)** | **MoCA, mean (SD)** |
| --- | --- | --- | --- | --- | --- | --- | --- |
|  |  |  |  |  |  |  |  |
| Thaler *et al* (2021), Israel | *GBA, LRRK2* | *GBA*-PD: 77  *LRRK2*-PD: 30  iPD: 31  *GBA*-NMC: 105  *LRRK2*-NMC:67  HC: 32 | *GBA*-PD: 66.3  *LRRK2*-PD: 60.0  iPD: 64.5  *GBA*-NMC: 35.8  *LRRK2*-NMC: 49.3  HC: 39.4 | *GBA*-PD: 68.43 (10.2)  *LRRK2*-PD: 65.21 (10.0)  iPD: 65.77 (10.4)  *GBA*-NMC: 54.88 (10.03)  *LRRK2*-NMC: 54.45 (12.46)  HC: 54.82 (10.03) | *GBA*-PD: 3.78 (2.6)  *LRRK2*-PD: 3.82 (2.7)  iPD: 3.69 (1.9) | *GBA*-PD: 40.54 (21.4)  *LRRK2*-PD: 35.87 (16.9)  iPD: 41.87 (15.7)  *GBA*-NMC: 5.94 (6.85)  *LRRK2*-NMC: 5.33 (4.29)  HC: 4.85 (4.76) | *GBA*-PD: 23.32 (4.4)  *LRRK2*-PD: 25.03 (4.0)  iPD: 23.71 (3.7)  *GBA*-NMC: 25.87 (3.13)  *LRRK2*-NMC: 26.05 (3.34)  HC: 27.39 (2.96) |
| Thaler *et al* (2018), Israel | *GBA* | *GBA*-PD group 1^&^: 48  *GBA*-PD group 2^&&^: 139  iPD: 152 | *GBA*-PD group 1^&^: 55.3  *GBA*-PD group 2^&&^: 59.7  iPD: 56.6 | *GBA*-PD group 1^&^: 64.15 (10.2)  *GBA*-PD group 2^&&^: 66.44 (10.0)  iPD: 63.23 (10.4) | *GBA*-PD group 1&: 6.47 (5.0)  *GBA*-PD group 2&&: 7.00 (6.1)  iPD: 4.91 (4.7) | *GBA*-PD group 1&: 26.84 (9.2)  *GBA*-PD group 2&&: 25.92 (12.4)  iPD: 15.59 (12.5) | *GBA*-PD group 1&: 23.84 (3.2)  *GBA*-PD group 2&&: 24.06 (3.7)  iPD: 25.00 (3.5) |
| Zhao *et al* (2020), China | *GBA, LRRK2, PRKN* | *GBA*-PD: 59  *LRRK2*-PD: 10  *PRKN*-PD: 83  iPD: 1544 | NA | *GBA*-PD: 49.63 (6.4)  *LRRK2*-PD: 55.30 (10.9)  *PRKN*-PD: 41.43 (11.5)  iPD: 52.25 (8.9) | *GBA*-PD: 5.78 (4.6)  *LRRK2*-PD: 7.10 (3.8)  *PRKN*-PD: 12.36 (9.2)  iPD: 6.24 (5.2) | *GBA*-PD: 28.73 (13.2)  *LRRK2*-PD: 31.30 (19.9)  *PRKN*-PD: 26.78 (16.4)  iPD: 27.49 (16.0) | NA |
| Canu *et al* (2021), Germany | *GBA* | *GBA*-PD: 10  iPD: 20 | *GBA*-PD: 30  iPD: 30 | *GBA*-PD: 62.1 (4.9)  iPD: 62.4 (5.2) | *GBA*-PD: 2.4 (0.7)  iPD: 2.4 (2.0) | *GBA*-PD: 15.4 (6.5)  iPD: 15.6 (4.3) | NA |
| Caminiti *et al* (2021), Italy | *GBA* | *GBA*-PD: 46  iPD: 58 | *GBA*-PD: 56.5  iPD: 56.9 | *GBA*-PD: 58.9 (9.6)  iPD: 47 (4.8) | *GBA*-PD: 1.5 (1.4)  iPD: 2.5 (3.2) | *GBA*-PD: 28.9 (10.2)  iPD: 21.7 (10.8) | *GBA*-PD: 26.9 (2.5)  iPD: 28.1 (2.3) |
| Moran *et al* (2021), USA | *GBA* | *GBA*-NMC: 30  HC: 49 | *GBA*-NMC: 63.3  HC: 75.5 | *GBA*-NMC: 53.93 (14.41)  HC: 59.90 (12.58) | - | *GBA*-NMC: 0.75 (1.19)  HC: 0.70 (1.36) | *GBA*-NMC: 27.60 (2.06)  HC:27.78 (1.81) |
| Simuni *et al* (2020), USA | *GBA, LRRK2* | *GBA*-NMC: 184  *LRRK2*-NMC: 208  HC: 194 | *GBA*-NMC: 38  *LRRK2*-NMC: 42  HC: 64 | *GBA*-NMC: 61.8 (6.9)  *LRRK2*-NMC: 61.6 (7.6)  HC: 60.8 (11.3) | - | *GBA*-NMC:2.5 (3.7)  *LRRK2*-NMC: 1.0 (2.1)  HC: 0.4 (1.0) | *GBA*-NMC: 26.8 (2.4)  *LRRK2*-NMC: 26.8 (2.4)  HC: 28.2 (1.1) |

**Table S3 continued**

| **Source (y), country** | **Gene variants** | **Sample size (n)** | **Sex (male, %)** | **Age at enrollment, mean (SD), y** | **Disease duration, mean (SD), y** | **UPDRS III, mean (SD)** | **MoCA, mean (SD)** |
| --- | --- | --- | --- | --- | --- | --- | --- |
|  |  |  |  |  |  |  |  |
| Avenali *et al* (2019), Italy | *GBA* | *GBA*-NMC: 16  HC: 16 | *GBA*-NMC: 56.3  HC: 50 | *GBA*-NMC: 59.0 (9.0)  HC: 56.5 (11.0) | - | *GBA*-NMC (baseline / follow-up): 0.94 (0.46) / 3.31 (1.05)  HC (baseline / follow-up): 0.44 (0.27) / 2.06 (0.77) | *GBA*-NMC (baseline / follow-up): 25.81 (0.7) / 27.19 (0.46)  HC (baseline / follow-up): 26.56 (0.50) / 28.06 (0.42) |
| Chahine *et al* (2018), USA | *GBA, LRRK2* | *GBA*-NMC: 38  *LRRK2*-NMC: 88  HC: 126 | *GBA*-NMC: 29  *LRRK2*-NMC: 36  HC: 66 | *GBA*-NMC: 63.6 (7.5)  *LRRK2*-NMC: 61.6 (7.1)  HC: 62.2 (7.3) | - | NA | NA |
| Beavan *et al* (2015), UK | *GBA* | *GBA*-NMC: 28  HC: 26 | *GBA*-NMC: 42.9  HC: 53.8 | *GBA*-NMC: 63.6 (10.6)  HC: 61.7 (11.2) | - | *GBA*-NMC (baseline / follow-up): 1.97 (3.44) / 4.50 (3.97)  HC (baseline / follow-up): 0.21 (0.87) / 0.92 (1.89) | *GBA*-NMC (baseline / follow-up): 25.55 (3.07) / 26.21 (3.02)  HC (baseline / follow-up): 27.32 (1.17) / 27.73 (1.33) |
| Ehrminger *et al* (2015), France | *LRRK2* | *LRRK2*-PD: 10  iPD: 20  *LRRK2*-NMC: 4  HC: 12 | *LRRK2*-PD: 70  iPD: 70  *LRRK2*-NMC: 50  HC: 50 | *LRRK2*-PD: 60.5 (11.4)  iPD: 65.9 (8.7)  *LRRK2*-NMC: 45.8 (15.5)  HC: 45.8 (11.2) | *LRRK2*-PD: 8.3 (3.1)  iPD: 9.0 (4.4) | NA | NA |
| Gaig *et al* (2014), USA | *LRRK2* | *LRRK2*-PD: 33  iPD: 33 | *LRRK2*-PD: 45.4  iPD: 45.4 | *LRRK2*-PD: 64.8 (11.4)  iPD: 65.1 (10.0) | *LRRK2*-PD: 9.2 (5.7)  iPD: 9.0 (6.1) | *LRRK2*-PD: 24.0 (14.0)  iPD: 19.9 (12.2) | NA |
| Trinh *et al* (2014), Canada | *LRRK2* | *LRRK2*-PD: 75  iPD: 118 | *LRRK2*-PD: 56  iPD: 53 | *LRRK2*-PD: 67.6 (12.6)  iPD: 66.6 (12.9) | NA | *LRRK2*-PD: 36.7 (16.4)  iPD: 33.3 (19.5) | *LRRK2*-PD: 21.7 (6.8)  iPD: 19.3 (8.5) |
| Marras *et, al* (2016), Canada | *LRRK2* | *LRRK2*-PD: 516  iPD: 790 | *LRRK2*-PD: 51.6  iPD: 58.9 | *LRRK2*-PD: 64.98 (11.5)  iPD: 65.70 (11.3) | *LRRK2*-PD: 9.43 (6.6)  iPD: 7.24 (6.0) | *LRRK2*-PD: 27.68 (28.60)  iPD: 24.55 (9.27) | *LRRK2*-PD: 24.03  iPD: 28.83 |
| Alcalay *et al* (2013), USA | *LRRK2* | *LRRK2*-PD: 97  iPD: 391 | *LRRK2*-PD: 48.5  iPD: 62.1 | *LRRK2*-PD: 68.6 (8.8)  iPD: 68.1 (10.3) | *LRRK2*-PD: 8.6 (7.2)  iPD: 6.1 (5.1) | *LRRK2*-PD: 19.8 (12.8)  iPD: 19.9 (11.3) | *LRRK2*-PD: 25.2 (2.9)  iPD: 24.7 (3.9) |

**Table S3 continued**

| **Source (y), country** | **Gene variants** | **Sample size (n)** | **Sex (male, %)** | **Age at enrollment, mean (SD), y** | **Disease duration, mean (SD), y** | **UPDRS III, mean (SD)** | **MoCA, mean (SD)** |
| --- | --- | --- | --- | --- | --- | --- | --- |
|  |  |  |  |  |  |  |  |
| Saunders-Pullman *et al* (2015), USA | *LRRK2* | *LRRK2*-PD: 142  iPD: 144  *LRRK2*-NMC: 117  HC: 113 | *LRRK2*-PD: 54.2  iPD: 64.6  *LRRK2*-NMC: 40.2  HC: 42.9 | *LRRK2*-PD: 67.2 (9.8)  iPD: 64.9 (11.3)  *LRRK2*-NMC: 53.3 (15.9)  HC: 52.8 (18.5) | *LRRK2*-PD: 10.0 (7.1)  iPD: 7.4 (7.5) | *LRRK2*-PD: 12.1 (13.1)  iPD: 20.1 (11.8)  *LRRK2*-NMC: 2.5 (3.5)  HC: 2.5 (3.5) | NA |
| Liang *et al* (2018), China | *LRRK2* | *LRRK2*-PD: 62  iPD: 623 | *LRRK2*-PD: 56.5  iPD: 59.1 | *LRRK2*-PD: 60.95 (13.3)  iPD: 61.86 (13.0) | NA | *LRRK2*-PD: 25.25 (14.2)  iPD: 28.64 (16.3) | NA |
| Li *et al* (2015), China | *LRRK2* | *LRRK2*-PD: 163  iPD: 1062 | *LRRK2*-PD: 60.5  iPD: 64.4 | *LRRK2*-PD: 60.34 (10.5)  iPD: 61.65 (10.5) | *LRRK2*-PD: 3.86 (3.6)  iPD: 4.25 (4.1) | *LRRK2*-PD: 60.5  iPD: 64.4 | NA |
| Sun *et al* (2016), China | *LRRK2* | *LRRK2*-PD: 76  iPD: 225 | *LRRK2*-PD: 48.68  iPD: 55.56 | *LRRK2*-PD: 62.16 (8.0)  iPD: 62.85 (8.5) | *LRRK2*-PD: 6.57 (4.2)  iPD: 5.73 (3.6) | *LRRK2*-PD: 24.55 (12.1)  iPD: 26.52 (12.6) | NA |
| Yang *et al* (2021), China | *LRRK2* | *LRRK2*-PD: 12  iPD: 47 | *LRRK2*-PD: 33.3  iPD: 48.9 | *LRRK2*-PD: 63 (7.92)  iPD: 59.91 (8.84) | *LRRK2*-PD: 5.17 (4.24)  iPD: 4.12 (3.10) | *LRRK2*-PD: 52.17 (26.29)  iPD: 36.55 (17.82) | NA |
| Cui *et al* (2021), China | *LRRK2* | *LRRK2*-PD: 79  iPD: 534 | *LRRK2*-PD: 53.2  iPD: 55.2 | *LRRK2*-PD: 63.11 (9.73)  iPD: 61.5 (10.56) | *LRRK2*-PD: 5.31 (3.64)  iPD: 5.06 (4.32) | *LRRK2*-PD: 23.70 (18.63)  iPD: 22.78 (5.08) | *LRRK2*-PD: 23.29 (5.79)  iPD: 22.78 (5.08) |
| Pont-Sunyer *et al* (2015), Spain | *LRRK2* | *LRRK2*-PD: 18  iPD: 19  *LRRK2*-NMC: 17  HC: 14 | *LRRK2*-PD: 50  iPD: 57.9  *LRRK2*-NMC: 47.7  HC: 50 | *LRRK2*-PD: 61.0 (11.2)  iPD: 63.1 (11.2)  *LRRK2*-NMC: 44.4 (12.3)  HC: 50.8 (16.0) | *LRRK2*-PD: 7.8 (5.8)  iPD: 7.3 (3.7) | *LRRK2*-PD: 14.4 (7.7)  iPD: 21.8 (10.8)  *LRRK2*-NMC: 1.1 (1.9)  HC: 0.2 (0.4) | *LRRK2*-PD: 24.2 (4.7)  iPD: 24.9 (2.4)  *LRRK2*-NMC: 26.3 (2.4)  HC: 27.4 (1.9) |
| Johansen *et al* (2011), Norway | *LRRK2* | *LRRK2*-NMC: 32  HC: 15 | *LRRK2*-NMC: 59.4  HC: 60 | *LRRK2*-NMC: 54.8 (14.9)  HC: 52.8 (13.8) | - | *LRRK2*-NMC: 5.7 (3.6)  HC: 3.2 (2.2) | NA |
| Pont-Sunyer *et al* (2017), Spain | *LRRK2* | *LRRK2*-NMC: 342  HC: 259 | *LRRK2*-NMC: 41.57  HC: 45.59 | *LRRK2*-NMC: 51.58 (15.6)  HC: 51.52 (16.4) | - | *LRRK2*-NMC: 2.88 (2.36)  HC: 1.64 (1.31) | *LRRK2*-NMC: 26.25 (25.91)  HC: 26.24 (25.78) |

**Table S3 continued**

| **Source (y), country** | **Gene variants** | **Sample size (n)** | **Sex (male, %)** | **Age at enrollment, mean (SD), y** | **Disease duration, mean (SD), y** | **UPDRS III, mean (SD)** | **MoCA, mean (SD)** |
| --- | --- | --- | --- | --- | --- | --- | --- |
|  |  |  |  |  |  |  |  |
| van den Heuve *et al* (2018), Canada | *LRRK2* | *LRRK2*-NMC: 11  HC: 26 | *LRRK2*-NMC: 55  HC: 46 | *LRRK2*-NMC: 61.4 (17.89)  HC: 55 (17.67) | - | *LRRK2*-NMC: 4.0 (4.4)  HC: 3.0 (3.0) | NA |
| Mirelman *et al* (2015), Israel, USA | *LRRK2* | *LRRK2*-NMC: 134  HC: 119 | *LRRK2*-NMC: 44.1  HC: 49.6 | *LRRK2*-NMC: 51.08 (17.61)  HC: 49.11 (16.51) | - | *LRRK2*-NMC: 1.70 (3.00)  HC: 1.65 (2.25) | *LRRK2*-NMC: 27.00 (3.00)  HC: 26.47 (2.63) |
| Mestre *et al* (2018), Canada | *LRRK2* | *LRRK2*-NMC: 52  HC: 90 | *LRRK2*-NMC: 53.8  HC: 43.3 | *LRRK2*-NMC: 49.2 (14.5)  HC: 47.9 (12.5) | - | *LRRK2*-NMC: 1.7 (2.8)  HC: 1.8 (3.1) | NA |
| Morgante *et al* (2016), Italy | *PRKN* | *PRKN*-PD: 22  iPD: 26 | *PRKN*-PD: 59.1  iPD: 65.4 | *PRKN*-PD: 52.26 (10.9)  iPD: 55.1 (8.5) | *PRKN*-PD: 21.29 (10.9)  iPD: 11.82 (9.8) | *PRKN*-PD: 18.90 (9.6)  iPD: 21.0 (8.5) | NA |
| Song *et al* (2020), China | *PRKN* | *PRKN*-PD: 53  iPD: 208 | *PRKN*-PD: 58.5  iPD: 64.4 | *PRKN*-PD: 35.6 (8.8)  iPD: 44.8 (8.8) | *PRKN*-PD: 9.0 (6.4)  iPD: 5.2 (6.4) | *PRKN*-PD^†^: 33.2 (13.8)  iPD^†^: 30.5 (15.0) | NA |
| Kim *et al* (2011), Korea | *PRKN* | *PRKN*-PD: 14  iPD: 64 | *PRKN*-PD: 57.1  iPD: 59.4 | *PRKN*-PD: 43.9 (10.4)  iPD: 46.7 (8.5) | *PRKN*-PD: 16.9 (8.1)  iPD: 11.0 (6.3) | NA | NA |
| Kagi *et al* (2010), Italy | *PRKN* | *PRKN*-PD: 16  iPD: 27 | *PRKN*-PD: 68.8  iPD: 55.6 | *PRKN*-PD: 46.1 (9.3)  iPD: 52.0 (6.3) | *PRKN*-PD: 21.0 (9.0)  iPD: 14.2 (6.2) | *PRKN*-PD: 33.5 (13.6)  iPD: 29 (12.4) | NA |
| Limousin *et al* (2009), France | *PRKN* | *PRKN*-PD: 11  iPD: 11 | *PRKN*-PD: 27.3  iPD: 27.3 | *PRKN*-PD: 49 (8)  iPD: 59 (6) | *PRKN*-PD: 20 (6)  iPD: 5 (4) | NA | NA |
| Zhou *et al* (2020), China | *PRKN* | *PRKN*-PD: 24  iPD: 24 | *PRKN*-PD: 50  iPD: 66.7 | *PRKN*-PD: 33.17 (6.74)  iPD: 35.25 (6.30) | *PRKN*-PD: 6.79 (6.45)  iPD: 2.60 (1.60) | *PRKN*-PD: 26.83 (10.15)  iPD: 24.38 (12.82) | NA |

* *GBA*-PD group 1: PD with GD-causing variants (L444P, N370S, R463C, G202R, R359S), ** *GBA*-PD group 2: PD with *GBA* E326K and T369M variants

† MDS-UPDRS part III in *off*-stage

# *GBA*-PD group 1: PD with GD severe variant (L444P, G202R, R359X, W184R, c.1265-1319del), ## *GBA*-PD group 2: PD with GD mild variant (N370S), ### *GBA*-PD group 3: PD with GD risk variant (E326K, T369M, T297S)

& *GBA*-PD group 1: PD with GD severe variant (84GG, IVS2+1, L444P, V394L, 370Rec), && *GBA*-PD group 2: PD with GD mild variant (N370S, R496H)

SD, standard deviation; UPDRS III, Unified Parkinson’s Disease Rating Scale Part III; MoCA, Montreal Cognitive Assessment; *GBA*, glucocerebrosidase gene; *LRRK2*, Leucine-rich repeat kinase 2 gene; *PRKN*, parkin gene; PD, Parkinson’s disease; iPD: idiopathic PD; NMC, non-manifesting carrier; HC, healthy control; NA: not applicable

**Table S4 Primary outcomes of included studies**

| **Source (y), country** | **Gene variants** | **Sample size (n)** | **Sleep disorder assessment tools** | **Sleep disorder outcome, n, mean (SD)** | | | |
| --- | --- | --- | --- | --- | --- | --- | --- |
|  |  |  |  | **RBD** | **EDS** | **RLS** | **PDSS** |
| Chen *et al* (2020), China | *GBA*, *LRRK2* | *GBA*-PD: 32  *LRRK2*-PD: 18  iPD: 376 | NA | GBA-PD: 12  LRRK2-PD: 7  iPD: 132 | - | - | - |
| Malek *et al* (2018), UK | *GBA* | *GBA*-PD group 1^*^: 40  *GBA*-PD group 2^**^: 93  iPD: 1480 | RBDSQ score > 4 | *GBA*-PD group 1: 17  *GBA*-PD group 2: 46  iPD: 639 | - | - | - |
| Simuni *et al* (2020), USA | *GBA, LRRK2* | *GBA*-PD*:* 78  *LRRK2*-PD: 157  iPD: 361 | RBDSQ score > 4  ESS | *GBA*-PD: 41, 5.3 (3.7)  *LRRK2*-PD: 46, 3.5 (2.3)  iPD: 151, 4.6 (3.0) | *GBA*-PD: 6.5 (4.2)  *LRRK2*-PD: 7.1 (4.7)  iPD: 6.7 (4.2) | - | - |
| Yahalom *et al* (2019), Israel | *GBA*  *LRRK2* | *GBA*-PD*:* 60  *LRRK2*-PD: 55  iPD: 72 | RBD1Q | *GBA*-PD: 40  *LRRK2*-PD: 9  iPD: 27 | - | - | - |
| Lerche *et al* (2020), Germany | *GBA* | *GBA*-PD group 1^#^: 10  *GBA*-PD group 2^##^: 8  *GBA*-PD group 3^###^: 27  iPD: 53 | NA | *GBA*-PD group 1: 6  *GBA*-PD group 2: 4  *GBA*-PD group 3: 16  iPD: 13 | - | - | - |
| Gan-Or *et al* (2015), Canada | *GBA* | *GBA*-PD: 19  iPD: 101 | RBDSQ score > 5 | *GBA*-PD: 9, 5.63 (3.7)  iPD: 24, 3.43 (2.9) | - | - | - |
| Bonner *et al* (2020), UK | *GBA* | *GBA*-PD: 15  iPD: 5 | NA | *GBA*-PD: 7  iPD: 15 | - | - | - |

**Table S4 Continued**

| **Source (y), country** | **Gene variants** | **Sample size (n)** | **Sleep disorder assessment tools** | **Sleep disorder outcome, n, mean (SD)** | | | |
| --- | --- | --- | --- | --- | --- | --- | --- |
|  |  |  |  | **RBD** | **EDS** | **RLS** | **PDSS** |
| Thaler *et al* (2021), Israel | *GBA, LRRK2* | *GBA*-PD: 77  *LRRK2*-PD: 30  iPD: 31  *GBA*-NMC: 105  *LRRK2*-NMC:67  HC: 32 | RBDSQ | *GBA*-PD: 3.84 (3.1)  *LRRK2*-PD: 3.06 (2.3)  iPD: 3.1 (3.1)  *GBA*-NMC: 1.95 (1.91)  *LRRK2*-NMC: 1.40 (1.46)  HC: 1.79 (1.45) | - | - | - |
| Thaler *et al* (2018), Israel | *GBA* | *GBA*-PD group 1^&^: 48  *GBA*-PD group 2^&&^: 139  iPD: 152 | RBDSQ | *GBA*-PD group 1^&^: 4.71 (3.3)  *GBA*-PD group 2^&&^: 4.13 (3.5)  iPD: 3.38 (2.8) | - | - | - |
| Zhao *et al* (2020), China | *GBA, LRRK2, PRKN* | *GBA*-PD: 59  *LRRK2*-PD: 10  *PRKN*-PD: 83  iPD: 1544 | RBDQ-HK, ESS | *GBA*-PD: 18.20 (18.6)  *LRRK2*-PD: 16.75 (23.3)  *PRKN*-PD: 9.85 (12.2)  iPD: 13.43 (16.0) | *GBA*-PD: 7.90 (6.6)  *LRRK2*-PD: 11.75 (7.3)  *PRKN*-PD: 6.73 (64)  iPD: 7.38 (6.1) | - | - |
| Canu *et al* (2021), Germany | *GBA* | *GBA*-PD: 10  iPD: 20 | RBDSQ | *GBA*-PD: 3.7 (3.6)  iPD: 2.3 (2.2) | - | - | - |
| Caminiti et al (2021), Italy | *GBA* | *GBA*-PD: 46  iPD: 58 | RBDSQ | *GBA*-PD: 4.4 (3.0)  iPD: 3.0 (2.3) |  |  |  |
| Moran *et al* (2021), USA | *GBA* | *GBA*-NMC: 30  HC: 49 | RBDSQ > 4 | *GBA*-NMC: 1, 2.00 (1.53)  HC: 3, 1.36 (1.72) | - | - | - |
| Simuni *et al* (2020), USA | *GBA, LRRK2* | *GBA*-NMC: 184  *LRRK2*-NMC: 208  HC: 194 | RBDSQ > 4, ESS > 9 | *GBA*-NMC: 38  *LRRK2*-NMC: 42  HC: 39 | *GBA*-NMC: 18  *LRRK2*-NMC: 22  HC: 23 | - | - |

**Table S4 Continued**

| **Source (y), country** | **Gene variants** | **Sample size (n)** | **Sleep disorder assessment tools** | **Sleep disorder outcome, n, mean (SD)** | | | |
| --- | --- | --- | --- | --- | --- | --- | --- |
|  |  |  |  | **RBD** | **EDS** | **RLS** | **PDSS** |
| Avenali *et al* (2019), Italy | *GBA* | *GBA*-NMC: 16  HC: 16 | RBDSQ | *GBA*-NMC (baseline / follow-up): 0.20 (0.77) / 3.00 (2.87)  HC (baseline / follow-up): 0.42 (0.76) / 1.50 (2.18) | - | - | - |
| Chahine *et al* (2018), USA | *GBA, LRRK2* | *GBA*-NMC: 38  *LRRK2*-NMC: 88  HC: 126 | RBDSQ > 4 | *GBA*-NMC: 7  *LRRK2*-NMC: 16  HC: 23 | - | - | - |
| Beavan *et al* (2015), UK | *GBA* | *GBA*-NMC: 28  HC: 26 | RBDSQ | *GBA*-NMC (baseline / follow-up): 0.10 (0.53) / 2.30 (2.12)  HC (baseline / follow-up): 0.25 (0.71) / 1.08 (1.53) | - | - | - |
| Ehrminger *et al* (2015) France | *LRRK2* | *LRRK2*-PD: 10  iPD: 20  *LRRK2*-NMC: 4  HC: 12 | V-PSG, ESS | *LRRK2*-PD: 0  iPD: 9 | *LRRK2*-PD: 11.0 (6.1)  iPD: 9.0 (4.9)  *LRRK2*-NMC: 9.5 (1.3)  HC: 6.5 (3.1) | - | - |
| Gaig *et al* (2014) USA | *LRRK2* | *LRRK2*-PD: 33  iPD: 33 | ICSD-2, ESS, PDSS | *LRRK2*-PD: 7  iPD: 14 | *LRRK2*-PD: 6, 8.1 (4.6)  iPD: 13, 9.1 (5.3) | - | *LRRK2*-PD: 116.4 (16.8)  iPD: 116.7 (22.3) |
| Trinh *et al* (2014) Canada | *LRRK2* | *LRRK2*-PD: 75  iPD: 118 | RBDSQ, ESS, RLS | *LRRK2*-PD: 12  iPD: 34 | *LRRK2*-PD: 5.35 (4.90)  iPD: 4.88 (5.19) | *LRRK2*-PD: 9  iPD: 15 | - |
| Marras *et, al* (2016), Canada | *LRRK2* | *LRRK2*-PD: 516  iPD: 790 | RBDSQ > 5, ESS > 10 | *LRRK2*-PD: 21  iPD: 25 | *LRRK2*-PD: 4  iPD: 28 | - | - |
| Alcalay *et al* (2013), USA | *LRRK2* | *LRRK2*-PD: 97  iPD: 391 | RBD1Q | *LRRK2*-PD: 22  iPD: 137 | - | - | - |

**Table S4 Continued**

| **Source (y), country** | **Gene variants** | **Sample size (n)** | **Sleep disorder assessment tools** | **Sleep disorder outcome, n, mean (SD)** | | | |
| --- | --- | --- | --- | --- | --- | --- | --- |
|  |  |  |  | **RBD** | **EDS** | **RLS** | **PDSS** |
| Saunders-Pullman *et al* (2015), USA | *LRRK2* | *LRRK2*-PD: 142  iPD: 144  *LRRK2*-NMC: 117  HC: 113 | RBDSQ^‡^ | *LRRK2*-PD: 28, 3.35 (2.25)  iPD: 44, 4.35 (3.74)  *LRRK2*-NMC: 7, 2.00 (1.50)  HC: 25, 2.36 (2.27) | - | - | - |
| Liang *et al* (2018), China | *LRRK2* | *LRRK2*-PD: 15  iPD: 118 | RBDQ-HK, ESS | *LRRK2*-PD: 6  iPD: 55 | *LRRK2*-PD: 4  iPD: 50 | - | - |
| Li *et al* (2015), China | *LRRK2* | *LRRK2*-PD: 163  iPD: 1062 | RBD1Q, ESS | *LRRK2*-PD: 46  iPD: 317 | *LRRK2*-PD: 68  iPD: 495 | *LRRK2*-PD: 59  iPD: 386 | - |
| Sun *et al* (2016) China | *LRRK2* | *LRRK2*-PD: 76  iPD: 225 | RBDSQ > 5 | *LRRK2*-PD: 34, 5.00 (3.2)  iPD: 64, 3.87 (2.9) | - | - | - |
| Yang *et al* (2021) China | *LRRK2* | *LRRK2*-PD: 12  iPD: 47 | RBD1Q | *LRRK2*-PD: 8  iPD: 11 | - | - | - |
| Cui *et al* (2021) China | *LRRK2* | *LRRK2*-PD: 79  iPD: 534 | RBDQ-HK, ESS | *LRRK2*-PD: 14.62 (16.37)  iPD: 13.87 (17.33) | *LRRK2*-PD: 5.47 (5.69)  iPD: 5.79 (5.88) | - | - |
| Pont-Sunyer *et al* (2015) Spain | *LRRK2* | *LRRK2*-PD: 18  iPD: 19  *LRRK2*-NMC: 17  HC: 14 | RBDSQ, ESS > 10, RLS, PDS | *LRRK2*-PD: 3.1 (2.2)  iPD: 3.7 (2.6)  *LRRK2*-NMC: 2.7 (2.7)  HC: 1.1 (1.9) | *LRRK2*-PD: 4, 7.8 (5.0)  iPD: 5, 8.3 (2.7)  *LRRK2*-NMC: 4, 7.3 (4.5)  HC: 0, 5.5 (2.2) | *LRRK2*-PD: 3  iPD: 3  *LRRK2*-NMC: 2  HC: 2 | *LRRK2*-PD: 8.3 (3.5)  iPD: 5.7 (3.6)  *LRRK2*-NMC: 6.3 (6.2)  HC: 3.4 (6.2) |
| Johansen *et al* (2011), Norway | *LRRK2* | *LRRK2*-NMC: 32  HC: 15 | RBD1Q, ESS | *LRRK2*-NMC: 1  HC: 0 | *LRRK2*-NMC: 2.3 (1.8)  HC: 2.5 (1.9) | - | - |
| Pont-Sunyer *et al* (2017), Spain | *LRRK2* | *LRRK2*-NMC: 169  HC: 104 | RBDSQ > 4, ESS > 9 | *LRRK2*-NMC: 20, 2.54 (1.67)  HC: 19, 2.53 (1.70) | *LRRK2*-NMC: 25, 4.82 (12.93)  HC: 15, 6.24 (4.53) | - | - |

**Table S4 Continued**

| **Source (y), country** |  | **Gene variants** | **Sample size (n)** | **Sleep disorder assessment tools** | **Sleep disorder outcome, n, mean (SD)** | | | |
| --- | --- | --- | --- | --- | --- | --- | --- | --- |
|  |  |  |  |  | **RBD** | **EDS** | **RLS** | **PDSS** |
| van den Heuve *et al* (2018), Canada |  | *LRRK2* | *LRRK2*-NMC: 11  HC: 26 | RBDSQ | *LRRK2*-NMC: 2.0 (2.2)  HC: 2.5 (2.0) | - | - | - |
| Mirelman *et al* (2015), Israel, USA |  | *LRRK2* | *LRRK2*-NMC: 134  HC: 119 | RBDSQ | *LRRK2*-NMC: 2.70 (1.50)  HC: 1.85 (1.50) | - | - | - |
| Mestre *et al* (2018), Canada |  | *LRRK2* | *LRRK2*-NMC: 52  HC: 90 | ESS | - | *LRRK2*-NMC: 4.6 (3.3)  HC: 6.3 (3.7) | - | - |
| Morgante *et al* (2016) Italy |  | *PRKN* | *PRKN*-PD: 21  iPD: 26 | RBD1Q | *PRKN*-PD: 6  iPD: 3 | - | - | - |
| Song *et al* (2020), China |  | *PRKN* | *PRKN*-PD: 53  iPD: 208 | RBDSQ, ESS | *PRKN*-PD: 11  iPD: 38 | *PRKN*-PD: 5.6 (4.4)  iPD: 5.6 (3.8) | - | - |
| Kim *et al* (2011), Korea |  | *PRKN* | *PRKN*-PD: 14  iPD: 64 | NMSQ | *PRKN*-PD: 7  iPD: 22 | *PRKN*-PD: 0  iPD: 3 | *PRKN*-PD: 1  iPD: 6 | - |
| Kagi *et al* (2010), Italy |  | *PRKN* | *PRKN*-PD: 16  iPD: 27 | NMSQ | *PRKN*-PD: 3  iPD: 14 | *PRKN*-PD: 1  iPD: 9 | *PRKN*-PD: 2  iPD: 13 | - |
| Limousin *et al* (2009), France |  | *PRKN* | *PRKN*-PD: 11  iPD: 11 | V-PSG, ESS | *PRKN*-PD: 1  iPD: 6 | *PRKN*-PD: 10 (3)  iPD: 12 (6) | *PRKN*-PD: 5  iPD: 0 | - |
| Zhou *et al* (2020), China |  | *PRKN* | *PRKN*-PD: 24  iPD: 24 | RBDSQ, ESS | *PRKN*-PD: 3.79 (2.27)  iPD: 3.30 (2.40) | *PRKN*-PD: 6.25 (3.50)  iPD: 3.26 (2.38) | - | - |

* *GBA*-PD group 1: PD with GD-causing variants (L444P, N370S, R463C, G202R, R359S); ** *GBA*-PD group 2: PD with *GBA* E326K and T369M variants

† MDS-UPDRS part III in *off*-stage

# *GBA*-PD group 1: PD with GD severe variant (L444P, G202R, R359X, W184R, c.1265-1319del); ## *GBA*-PD group 2: PD with GD mild variant (N370S); ### *GBA*-PD group 3: PD with GD risk variant (E326K, T369M, T297S)

& *GBA*-PD group 1: PD with GD severe variant (84GG, IVS2+1, L444P, V394L, 370Rec), && *GBA*-PD group 2: PD with GD mild variant (N370S, R496H)

‡ RBD+, RBDSQ score > 4 (NMC) and RBDSQ score > 5 (PD)

SD, standard deviation; PD, Parkinson’s diseasea; iPD: idiopathic PD; NMC, non-manifesting carrier; HC, healthy control; RBD, rapid eye movement (REM) sleep behavior disorder; RBDSQ, RBD screening questionnaire; ESS, Epworth Sleepiness Scale; RBD1Q, RBD Single-Question Screen; PDSS, Parkinson Disease Sleep Scale; RLS, restless legs syndrome; RBDQ-HK, RBD questionnaire-Hong Kong; NMSQ, PD nonmotor symptoms questionnaire; V-PSG, video-polysomnography; ICSD-2, International Classification of Sleep Disorders, Revised: Diagnostic and Coding Manual; NA: not applicable

**Table S5 Results of meta-regression in patients with gene variants**

| **Gene variant** | **Covariant** | **No. of sources** | **Tau^2^** | **Adjusted R^2^ (%)** | **95% CI** | ***P* value** |
| --- | --- | --- | --- | --- | --- | --- |
| **RBD in PD patients with / without gene variants** | | | | | | |
| *GBA* | Age | 6 | 0.140 | -80.86 | 0.93-1.11 | 0.604 |
|  | Sex (male, %) | 6 | 0.136 | -75.29 | 0.95-1.09 | 0.439 |
|  | Disease duration | 5 | 0.022 | 66.34 | 0.90-1.24 | 0.348 |
|  | UPDRS III | 4 | 0.157 | -30.46 | 0.83-1.28 | 0.565 |
|  | MoCA | 4 | 0.135 | 8.66 | 0.18-2.56 | 0.336 |
| *LRRK2* G2385R | Age | 4 | 0 | 100.00 | 0.84-3.37 | 0.084 |
|  | Sex (male, %) | 4 | 0 | 100.00 | 0.85-1.02 | 0.077 |
|  | Disease duration | 3 | 1.308 | -114.24 | 0.00-3060.32 | 0.721 |
|  | UPDRS III | 4 | 1.003 | -119.43 | 0.87-1.17 | 0.867 |
| *PRKN* | Age | 5 | 1.513 | -95.60 | 0.68-1.41 | 0.884 |
|  | Sex (male, %) | 5 | 0.992 | -28.24 | 0.89-1.23 | 0.447 |
|  | Disease duration | 5 | 1.360 | -75.86 | 0.62-1.43 | 0.657 |
|  | UPDRS III | 3 | 0.915 | 7.01 | 0.26-3.23 | 0.458 |
| **RBD in asymptomatic patients with gene variants and HC** | | | | | | |
| *LRRK2* G2019S | Age | 6 | 0.137 | -20.86 | 0.88-1.08 | 0.533 |
|  | Sex (male, %) | 6 | 0.130 | -14.26 | 0.87-1.07 | 0.410 |
|  | UPDRS III | 6 | 0.11 | 2.96 | 0.65-1.21 | 0.337 |
|  | MoCA | 4 | 0 | 100.00 | 0.62-7.47 | 0.118 |
| **EDS in PD patients with / without gene variants** | | | | | | |
| *PRKN* | Age | 4 | 0.112 | 52.26 | 0.78-1.11 | 0.229 |
|  | Sex (male, %) | 3 | 0.699 | -78.83 | 0.62-1.69 | 0.682 |
|  | Disease duration | 4 | 0.105 | 54..95 | 0.73-1.15 | 0.239 |
|  | UPDRS III | 3 | 0.561 | -103.29 | 0.14-6.12 | 0.73 |
| **EDS in asymptomatic patients with gene variants and HC** | | | | | | |
| *LRRK2* | Age | 4 | 0.133 | -72.62 | 0.81-1.33 | 0.563 |
|  | Sex (male, %) | 4 | 0.111 | -44.29 | 0.84-1.14 | 0.589 |
|  | UPDRS III | 4 | 0.087 | -13.62 | 0.66-1.84 | 0.484 |
| **RLS in PD patients with / without gene variants** | | | | | | |
| *PRKN* | Age | 3 | 4.853 | -18.48 | 0.00-17577.59 | 0.525 |
| *LRRK2* | Sex (male, %) | 3 | 0 | 100.00 | 0.52-1.53 | 0.222 |
| G2019S | duration | 3 | 10.39 | -153.64 | 0.00-1454560 | 0.955 |

Abbreviations: OR, odds ratio; SMD, standard mean difference; RBD, rapid eye movement (REM) sleep behavior disorder; EDS, excessive daytime sleepiness; RLS, restless legs syndrome; PDSS, Parkinson Disease Sleep Scale; *GBA*, glucocerebrosidase gene; *LRRK2*, Leucine-rich repeat kinase 2 gene; *PRKN*, parkin gene; HC, healthy control

**References**

1. Hua P, Cui C, Chen Y, Yao Y, Yu CY, Xu LG, et al. Thyrotroph embryonic factor polymorphism predicts faster progression of Parkinson's disease in a longitudinal study. J Integr Neurosci. 2021;20(1):95-101.

2. Lin CY, Yu RL, Wu RM, Tan CH. Effect of ALDH2 on Sleep Disturbances in Patients with Parkinson's Disease. Sci Rep. 2019;9(1):18950.

3. Li J, Ruskey JA, Arnulf I, Dauvilliers Y, Hu MTM, Högl B, et al. Full sequencing and haplotype analysis of MAPT in Parkinson's disease and rapid eye movement sleep behavior disorder. Mov Disord. 2018;33(6):1016-20.

4. Lou F, Li M, Luo X, Ren Y. CLOCK 3111T/C Variant Correlates with Motor Fluctuation and Sleep Disorders in Chinese Patients with Parkinson's Disease. Parkinsons Dis. 2018;2018:4670380.

5. Mohtashami S, He Q, Ruskey JA, Zhou S, Dion PA, Allen RP, et al. TOX3 Variants Are Involved in Restless Legs Syndrome and Parkinson's Disease with Opposite Effects. J Mol Neurosci. 2018;64(3):341-5.

6. Gao L, Díaz-Corrales FJ, Carrillo F, Díaz-Martín J, Caceres-Redondo MT, Carballo M, et al. Brain-derived neurotrophic factor G196A polymorphism and clinical features in Parkinson's disease. Acta Neurol Scand. 2010;122(1):41-5.

7. Rissling I, Frauscher B, Kronenberg F, Tafti M, Stiasny-Kolster K, Robyr AC, et al. Daytime sleepiness and the COMT val158met polymorphism in patients with Parkinson disease. Sleep. 2006;29(1):108-11.

8. Frauscher B, Högl B, Maret S, Wolf E, Brandauer E, Wenning GK, et al. Association of daytime sleepiness with COMT polymorphism in patients with parkinson disease: a pilot study. Sleep. 2004;27(4):733-6.

9. Zhang B, Cui C, Yu H, Li G. Association between ZNF184 and symptoms of Parkinson's disease in southern Chinese. Neurol Sci. 2020;41(8):2121-6.

10. Kim R, Park S, Yoo D, Jun JS, Jeon B. Impact of the apolipoprotein E ε4 allele on early Parkinson's disease progression. Parkinsonism Relat Disord. 2021;83:66-70.

11. Deutschlander AB, Konno T, Soto-Beasley AI, Walton RL, van Gerpen JA, Uitti RJ, et al. Association of MAPT subhaplotypes with clinical and demographic features in Parkinson's disease. Ann Clin Transl Neurol. 2020;7(9):1557-63.

12. Wu H, Li H, Shi Z, Tang J, Mei S, Ai T, et al. Association between NMD3 and symptoms of Parkinson's disease in Chinese patients. BMC Neurol. 2020;20(1):19.

**Figure S1 Severity of EDS in PD patients with and without *GBA* variants**


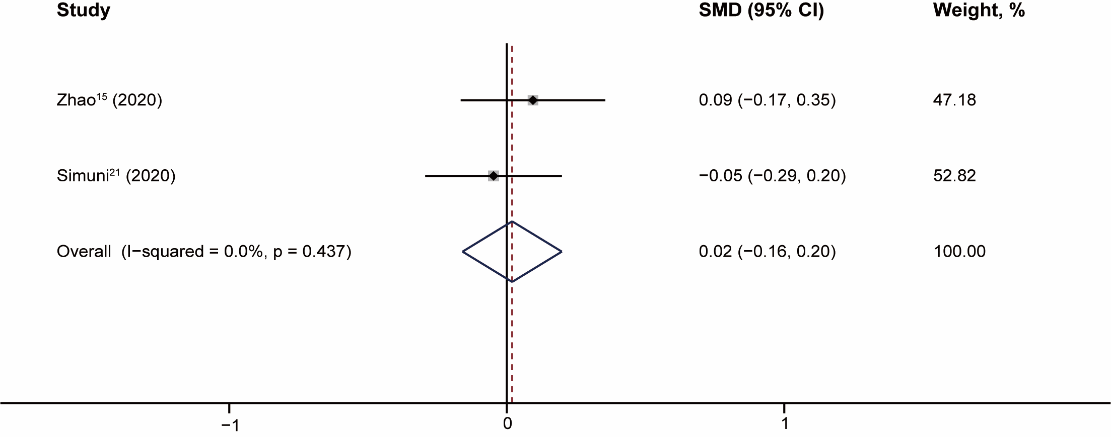


**Figure S2 Risk (a) and severity (b) of EDS in PD patients with and without *LRRK2* variants, severity of EDS in PD patients with and without *LRRK2* G2019S variants (c)**

**
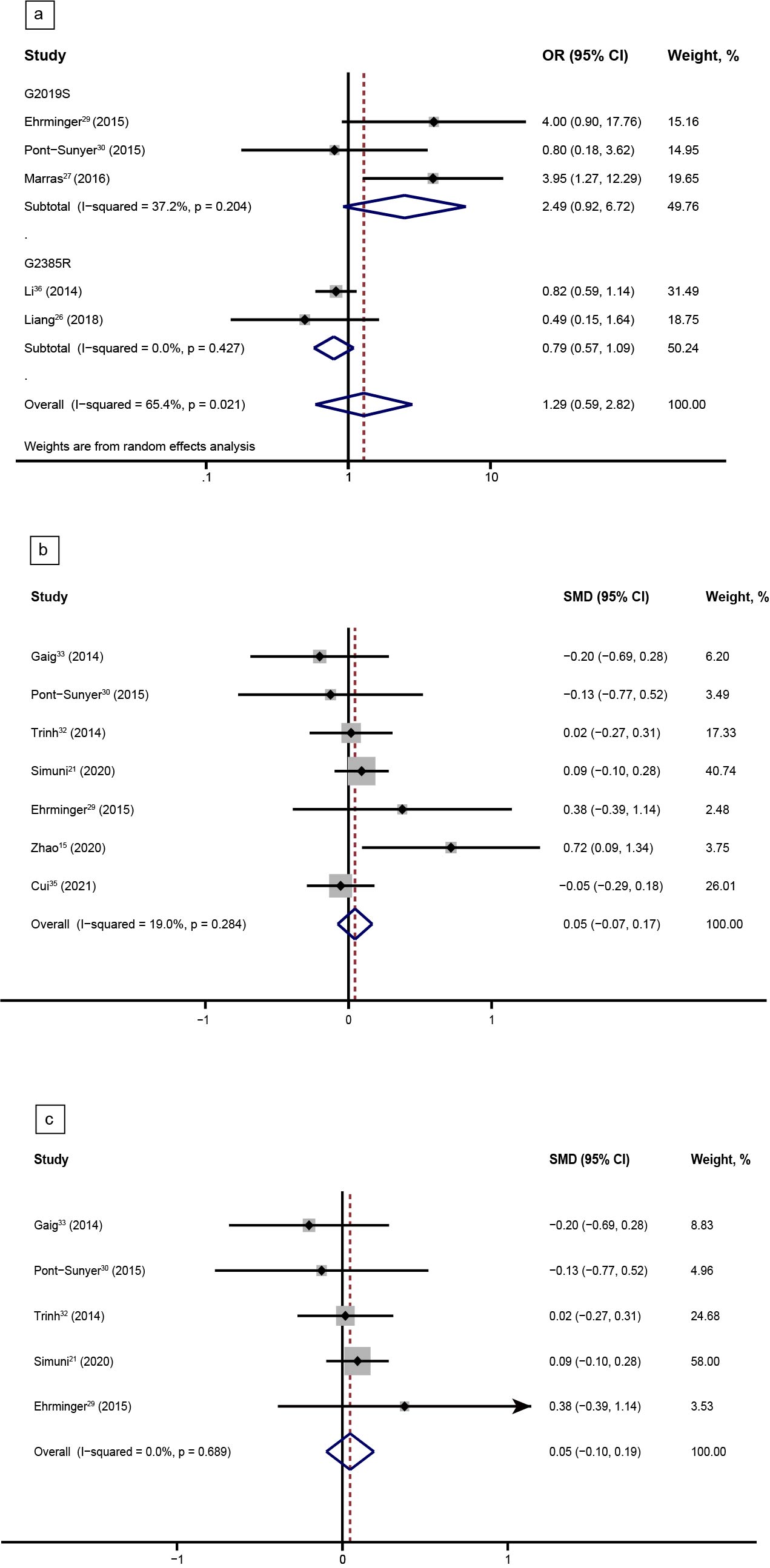
**

**Figure S3 PDSS score (a) and risk of RLS (b) in PD patients with and without *LRRK2* variants**

**
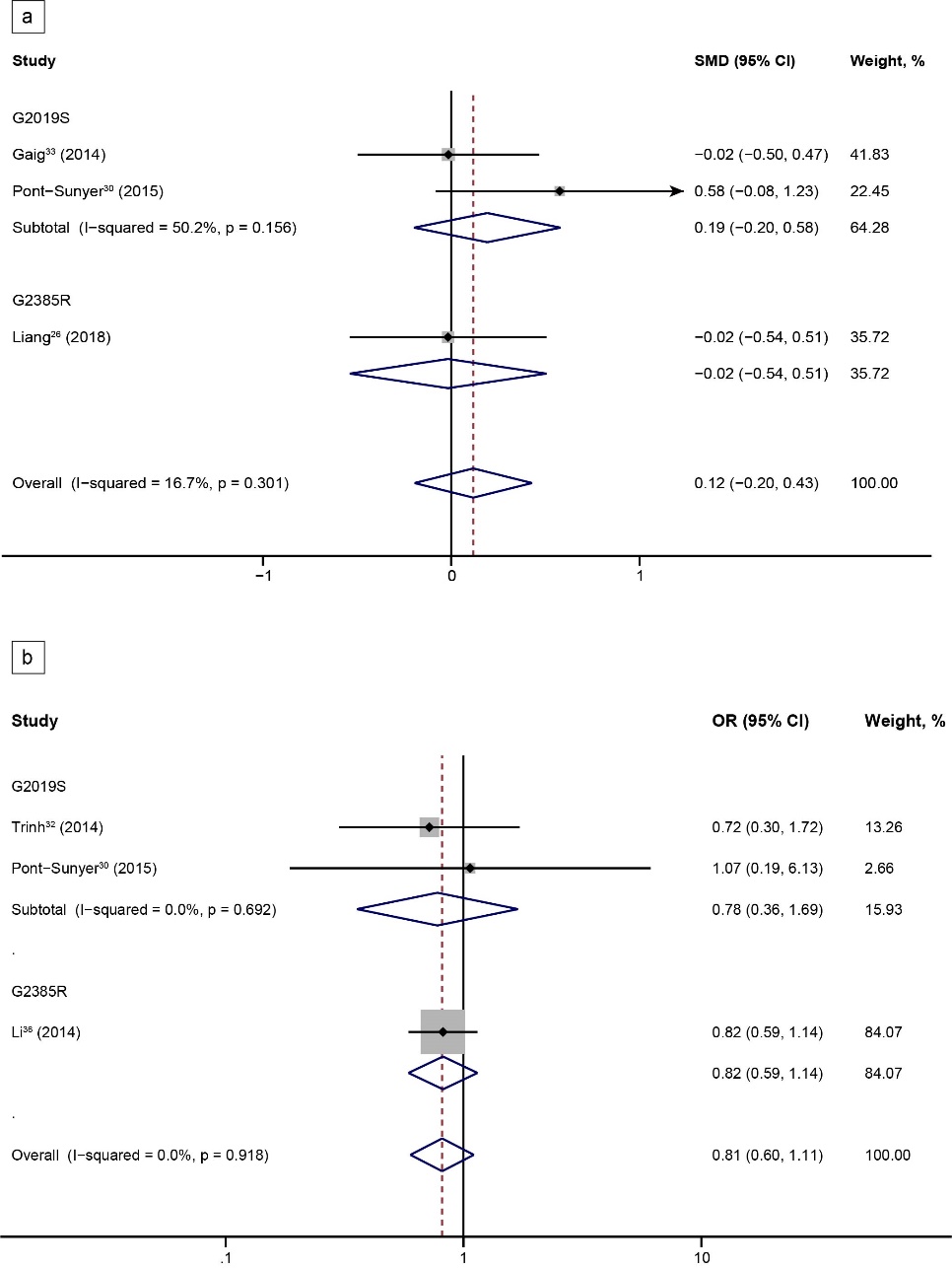
**

**Figure S4 Funnel plots for the risk of RBD in PD patients with *LRRK2* variants**


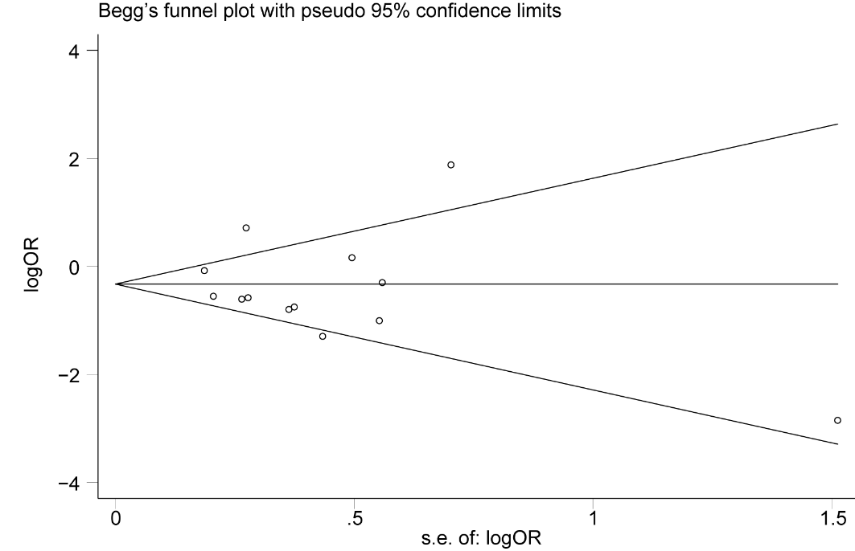


**Figure S5 Risk (a) and severity (b) of RBD in PD patients with *PRKN* variants**

**
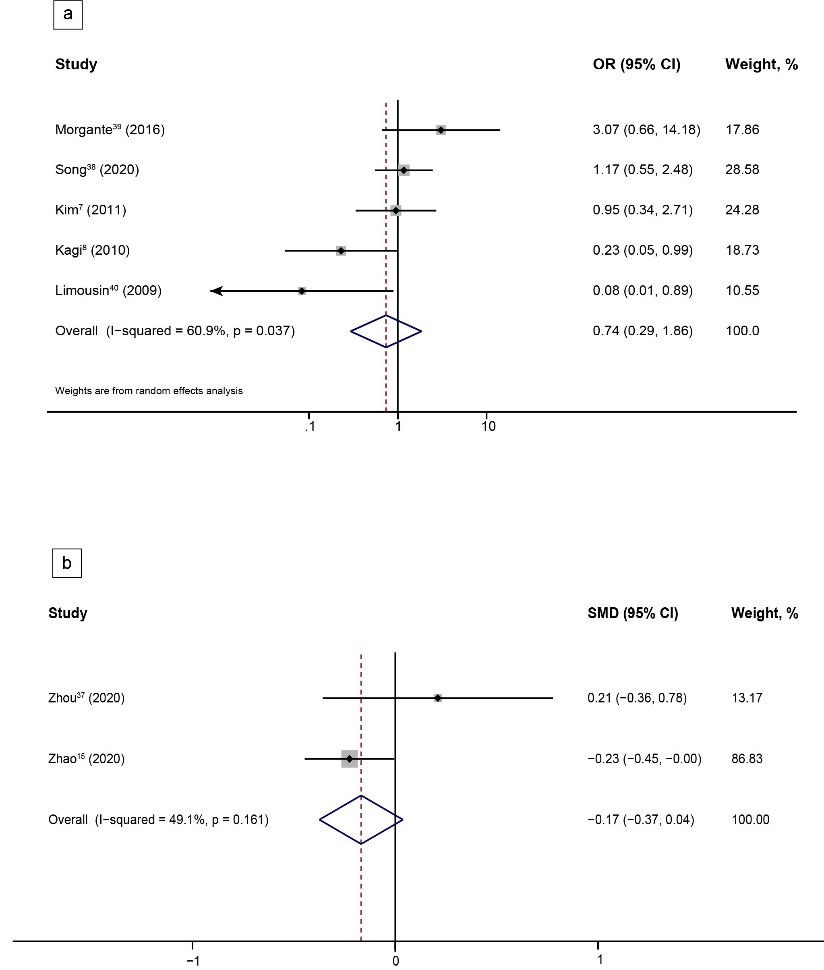
**

**Figure S6 Risk (a) and severity (b) of EDS in PD patients with *PRKN* variants**

**
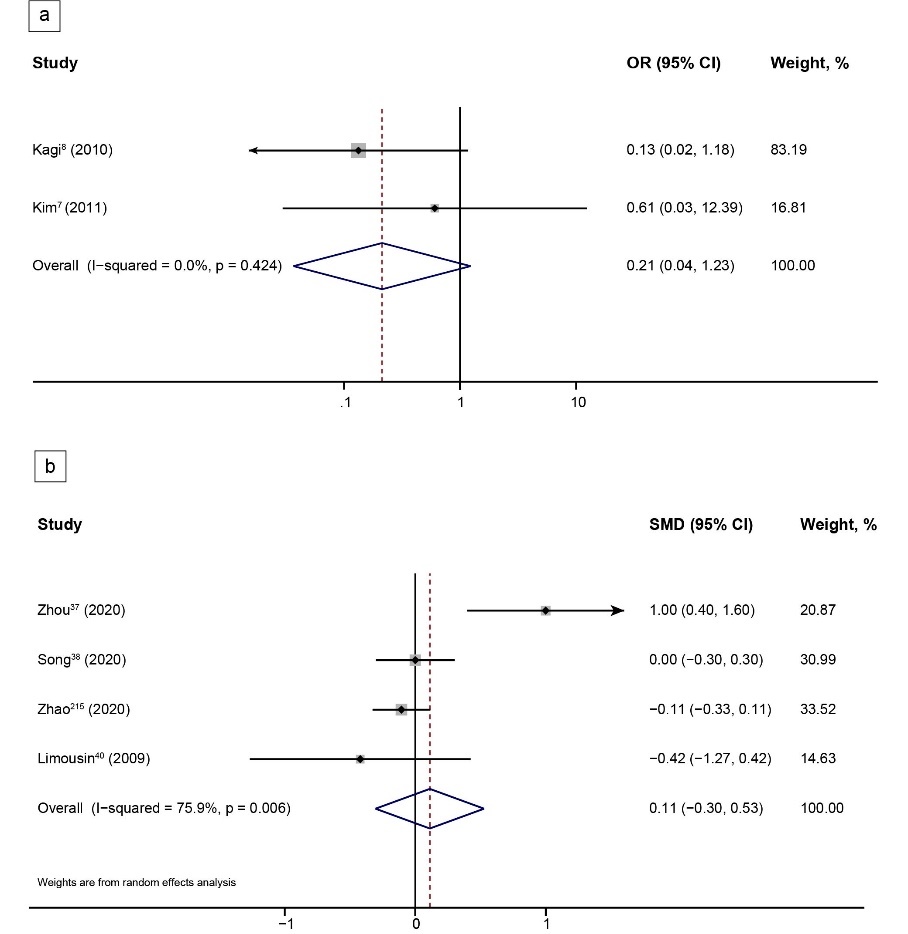
**

**Figure S7 Risk of RLS in PD patients with and without *PRKN* variants**

**
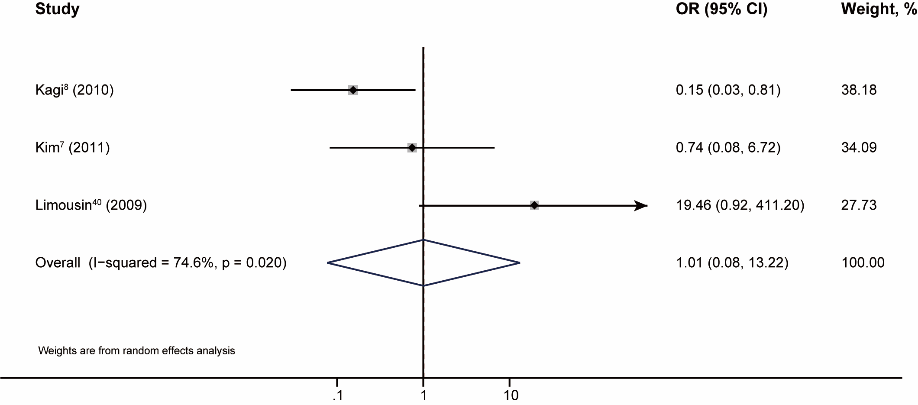
**

**
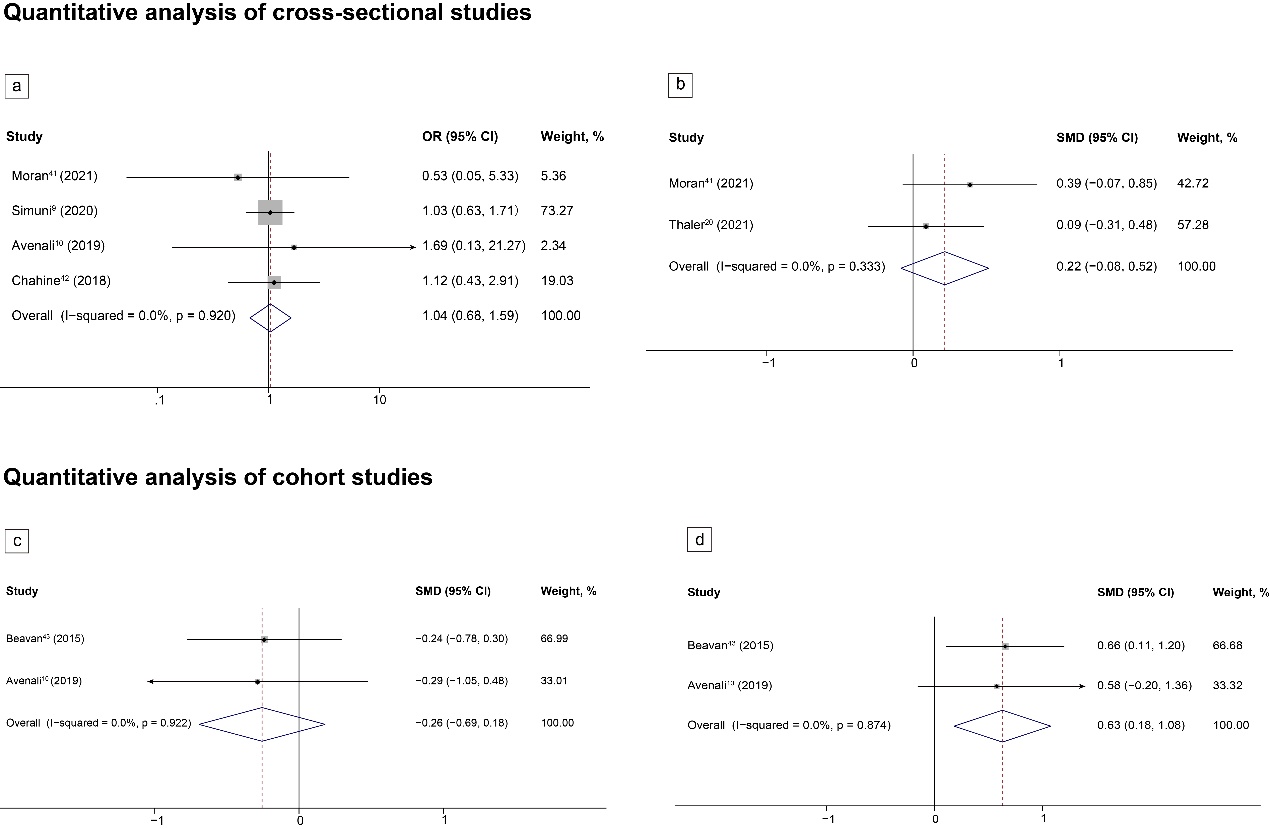
Figure S8 Risk (a) and severity (b) of RBD in asymptomatic carriers with *GBA* variant and HCs**

Forest plots display meta-analysis results of cross-sectional studies on the risk (a) and severity (b) in asymptomatic carriers with *GBA* variants compared with non-carrier HC and the meta-analysis results of longitudinal cohort studies on the severities in asymptomatic carriers with *GBA* variants compared with non-carrier HC (c, baseline; d, follow-up).

**
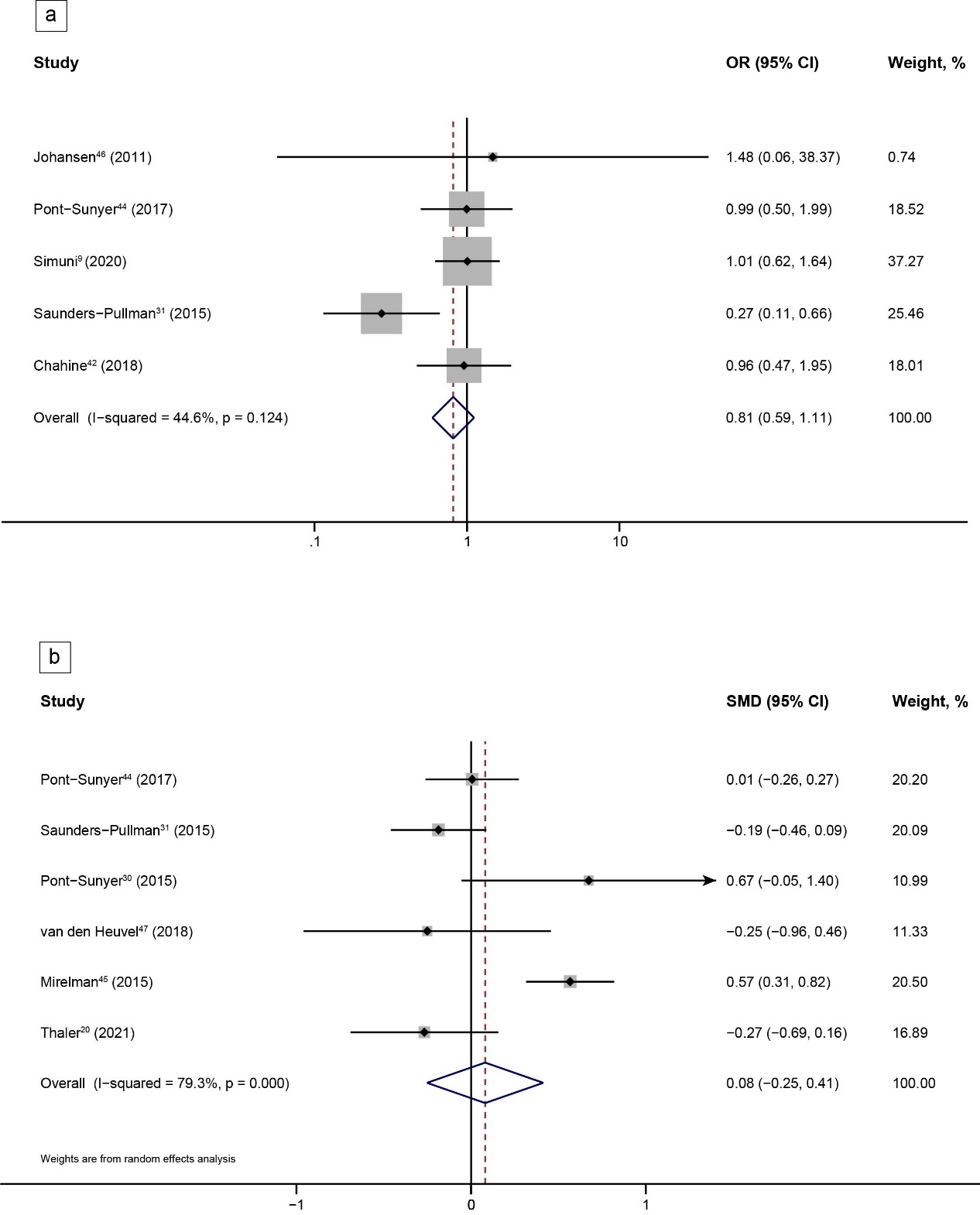
Figure S9 Risk (a) and severity (b) of RBD in asymptomatic carriers with *LRRK2* G2019S and HCs**

**Figure S10 Risk (a) and severity (b) of EDS in asymptomatic carriers with *LRRK2* G2019S and HCs**

**
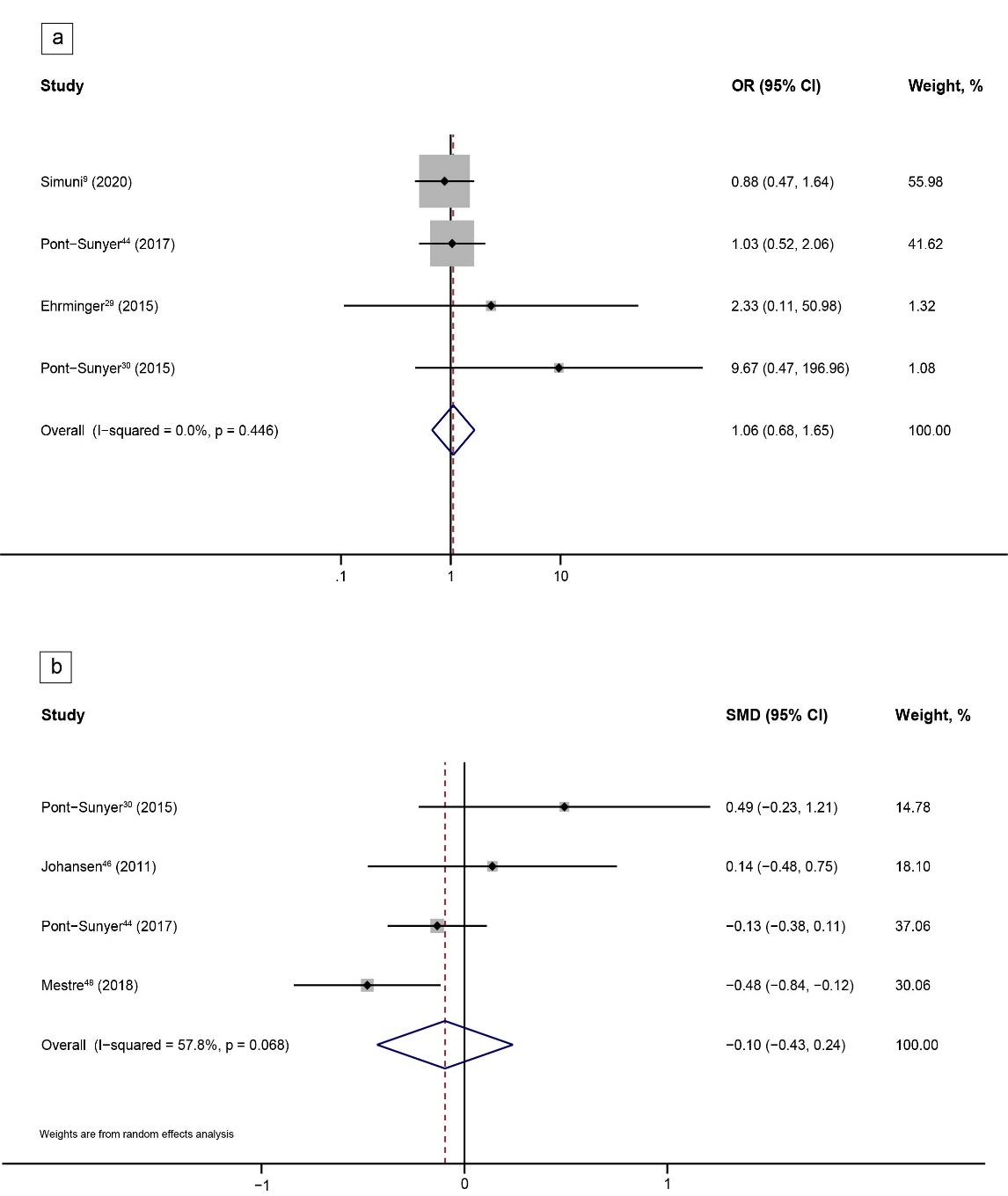
**
